# High resolution lipoprotein subfraction profiling identifies obesity associated distributional changes in LDL and HDL subfractions and particle ratios

**DOI:** 10.1038/s41366-026-02172-6

**Published:** 2026-08-13

**Authors:** Novia Minaee, Reika Masuda, Philipp Nitschke, Drew Hall, Jennie Hui, Michael L. Hunter, Leon Flicker, Graeme J. Hankey, Osvaldo P. Almeida, Dick C. Chan, Gerald F. Watts, Muhammad Saeed Ahmad, Huda M. Alkreathy, Zoheir Abdullah Dhamanouri, Hartmut Schäfer, Manfred Spraul, Claire Cannet, John P. Beilby, Bu B. Yeap, Oscar Millet, Julien Wist, Jeremy K. Nicholson, Samantha Lodge, Elaine Holmes

**Affiliations:** 1 Centre for Computational and Systems Medicine, Health Futures Institute, Murdoch University, Harry Perkins Building, Perth, WA Australia; 2 Busselton Population Medical Research Institute, Busselton, WA Australia; 3 Department of Diagnostic Genomics, PathWest Laboratory Medicine, Queen Elizabeth II Medical Centre, Perth, WA Australia; 4 School of Population and Global Health, University of Western Australia, Perth, WA Australia; 5 Medical School, University of Western Australia, Perth, WA Australia; 6 Perron Institute for Neurological and Translational Science, Nedlands, WA Australia; 7 Institute for Health Research, University of Notre Dame Australia, Fremantle, WA Australia; 8 School of Population Health, Curtin University, Perth, WA Australia; 9 Department of Cardiology and Internal Medicine, Royal Perth Hospital, Perth, WA Australia; 10 Department of Veterinary Medicine, University of Cambridge, Cambridge, UK; 11 Drug Metabolism Unit, King Fahad Medical Research Center, King Abdulaziz University, Jeddah, Saudi Arabia; 12 Department of Clinical Pharmacology, Faculty of Medicine, King Abdulaziz University, Jeddah, Saudi Arabia; 13 Bruker Biospin GmbH & Co. Kg, Ettlingen, Germany; 14 School of Biomedical Sciences, University of Western Australia, Perth, WA Australia; 15 Department of Endocrinology and Diabetes, Fiona Stanley Hospital, Perth, WA Australia; 16 Precision Medicine and Metabolism Laboratory, CIC bioGUNE, Parque Tecnológico de Bizkaia, Derio, Spain; 17 Chemistry Department, Universidad del Valle, Cali, Colombia; 18 Institute of Global Health Innovation, Imperial College London, London, UK; 19 School of Medicine, The Hong Kong University of Science and Technology, Hong Kong, China; 20 Department of Metabolism, Digestion and Reproduction, Faculty of Medicine, Imperial College London, London, UK

**Keywords:** Risk factors, Lipidomics

## Abstract

**Background:**

Obesity is associated with adverse alterations in lipoprotein profiles and increased cardiometabolic risk, yet standard clinical lipid measures provide limited resolution of the underlying lipoprotein structure. Here, we perform a population-level characterisation of the impact of obesity on the high-resolution blood lipoprotein subfraction profile.

**Methods:**

Lipoprotein profiles were investigated in an Australian cohort (*n* = 1806) stratified by Body Mass Index (BMI) (healthy weight, overweight, obese). The composition and particle number of lipoproteins and their subfractions were quantified (*n* = 112 parameters) using ^1^H NMR spectroscopy. Associations between BMI, lipoprotein subfractions, and particle number ratios were evaluated. Key BMI-associated ratios were validated in a second Australian cohort (*n* = 1313)

**Results:**

Participants with obesity had significantly higher particle concentrations of VLDL and IDL and lower HDLs, while total LDL concentration was similar between the BMI categories. Notably, obesity was associated with a marked redistribution of LDL subfractions, with a shift from larger, buoyant LDL1–3 particles to smaller, dense LDL4–6 particles, accompanied by a similar shift in HDL subfractions, with proportionally lower concentrations of HDL1–3 particles in the high BMI group. An inverse association between BMI was found for two LDL particle number ratios (LDL1–3:LDL4–6 and LDL2:LDL5) and one HDL ratio (HDL1:HDL4), which demonstrated good discriminatory power for obesity status (AUROCs 0.70–0.74).

**Conclusions:**

The pattern of lipoprotein subfraction redistribution in participants with obesity was consistent across cohorts. These subfractional changes offer enhanced resolution compared with conventional lipid and lipoprotein measures and may contribute to improved cardiovascular risk stratification in individuals with obesity.

## Introduction

Obesity is a major and increasing global health issue, with the World Obesity Federation predicting that by 2035, 51% of the global population will be living with overweight or obesity, with an estimated $4.32 trillion annual healthcare burden. Multiple metabolic disorders including dyslipidemia [1], type 2 diabetes [2] and cardiovascular disease [3] are associated with obesity. Despite its widespread impact, the molecular mechanisms linking obesity to these conditions remain incompletely understood. Plasma lipoproteins, the particles that transport essential lipids (cholesterol, triglycerides, phospholipids) coupled with apolipoproteins, are key regulators of metabolic homeostasis and are systematically altered in obesity and obesity related co-morbidities [4]. Obesity-induced shifts in lipoprotein composition and distribution, therefore, could be critical to understanding mechanistic drivers of metabolic dysfunction. Establishing whether these alterations are consistent across diverse populations is essential for refining global risk assessment and developing targeted interventions to address the rising burden of obesity-related diseases [5]. In an obesogenic environment, the plasma lipoproteome is characterised by elevated levels of triglyceride-rich very low-density lipoprotein (VLDL), reduced high-density lipoprotein (HDL)-cholesterol and near-normal low density lipoprotein (LDL)-cholesterol [6].

In the clinic, routine measures of blood lipids used to assess an individual’s risk of developing atherosclerotic cardiovascular disease (ASCVD) include total cholesterol, LDL and HDL cholesterol and triglycerides, with apolipoproteins B100 and A1 being monitored less frequently [7]. VLDL is not routinely measured clinically, since it generally requires time-consuming measurement using ultracentrifugation, gradient gel electrophoresis or ion mobility analysis [8] and typically triglyceride concentrations serve as an indirect measure of VLDL levels [9].

^1^H Nuclear Magnetic Resonance (NMR) spectroscopy can be used to reproducibly measure the lipoprotein subfractions of a blood plasma or serum sample using multivariate regression models calibrated against reference concentrations of lipoprotein subclasses determined by sequential density gradient ultracentrifugation to quantitatively resolve individual lipoprotein classes and subfractions within complex plasma matrices [10]. Using the Bruker IVDr Lipoprotein Subclass Analysis 112 lipoprotein parameters including particle number, apolipoprotein concentrations and lipoprotein ratios, for 4 classes of lipoprotein, VLDL (0.950–1.006 kg/L), intermediate density lipoprotein (IDL; 1.006–1.019 kg/L), LDL (1.019–1.063 kg/L), HDL (1.063–1.210 kg/L) can be generated in a 4 min experiment [11, 12]. NMR lipoprotein profiles have been used to characterise lipoprotein abnormalities associated with ASCVD [13], diabetes [2], inflammatory bowel disease [14] and acute inflammatory conditions such as SARS-CoV-2 infection [15].

This expanded panel of lipoproteins reflects more nuanced and deeper relationships between the various lipoproteins [9] and their sub-particle distributions in obesity, which may be valuable for developing targeted therapeutic strategies for obesity and related comorbidities. For example, increased levels of total cholesterol and LDL cholesterol are known to increase an individual’s risk of ASCVD. However small, dense LDL particles, which are characteristic of dyslipidaemia in obese individuals, predict increased risk of ASCVD even when total LDL cholesterol concentrations are within normal limits [16]. Similarly, HDL subfractions possess distinct structural and functional properties and emerging evidence suggests that specific subfractions, particularly smaller, denser particles, may exhibit pro-atherogenic characteristics, challenging the traditional view of HDL as uniformly cardioprotective [17].

While obesity is associated with adverse lipid profiles, the contribution of specific lipoprotein subfractions to this risk is unclear and underexplored in population studies. Current clinical lipid testing overlooks distributional differences that may better reflect cardiometabolic risk. We address this gap by analysing detailed lipoprotein subfraction distributions and particle number ratios in a large cohort of Australian adults. We aimed to assess the use of an expanded NMR lipoprotein profile and specifically the contribution of the different density subfractions within LDL and HDL classes in characterising BMI associated changes and potential cardiometabolic risk in a predominantly Caucasian population.

## Materials and methods

### Participant enrolment and sample collection in two cohort studies

#### The Busselton Healthy Ageing Study (Australian Population Cohort)

The Busselton Healthy Ageing Study (BHAS) is a community-based, prospective cohort study involving adults born between 1946 and 1964, resident in the Busselton Shire, Western Australia [18]. A total of 1806 participants aged between 45.5 and 67.2 years (27.9% healthy weight, 45.8% people with overweight and 26.3% living with obesity) provided overnight fasted plasma samples between 2010 and 2015, which were collected via venipuncture using the K2 EDTA BD Vacutainer system and fractions stored at −80°C. Height, weight and blood pressure were measured and routine haematological and biochemical testing on blood samples was performed as previously described [18]. Medical history and medication use including statin use were recorded. For this study participants with diabetes were excluded. Cohort demographics can be found in Supplementary Table S1.

#### The Health In Men Study (validation cohort)

The Health In Men Study (HIMS) is a population based randomized trial of screening for abdominal aortic aneurysms conducted in Perth, Western Australia in 1996–1999. Detailed descriptions of recruitment, participation rates, and mortality differences have been published previously [19]. In this study plasma samples from 1313 participants were collected between May 2011 and May 2013, when participants were aged between 79 and 97 years old.

#### ^1^H NMR sample preparation

Samples were stored at −80°C until the day of analysis, then defrosted at room temperature for 30 min. Each sample was prepared as a 1:1 ratio with phosphate buffer (75 mM Na_2_HPO_4_, 2 mM NaN_3_, 4.6 mM sodium trimethylsilyl propionate-[2,2,3,3-^2^H_4_] (TSP) in H_2_O/D_2_O 4:1, pH 7.4 ± 0.1), mixed and 600 μL added into 5 mm SampleJet^TM^ NMR tubes. Samples were maintained at 5°C inside the SampleJet^TM^ automatic sample changer until measurement.

### ^1^H NMR spectroscopy data acquisition and processing parameters

NMR spectroscopic analyses were performed on 600 MHz Bruker Avance III HD spectrometers, equipped with a 5 mm BBI probe and fitted with the Bruker SampleJet^TM^ robot cooling system set to 5°C. A full quantitative calibration was completed prior to sample measurement. For each sample, a single one-dimensional (1D) NMR experiment was performed at 310 K using the Bruker In Vitro Diagnostics research (IVDr) with solvent signal presaturation (32 scans, 98k data points, spectral width of 30 ppm, and experiment time of 4 min). A total of 112 lipoprotein parameters were measured using the Bruker IVDr Lipoprotein Subclass Analysis (B.I.-LISA) method whereby the –(CH_2_)n at δ = 1.25 and -CH_3_ at δ = 0.80 peaks of the spectrum, after normalization to the Bruker QuantRef^TM^ manager within Topspin, were quantified using a PLS-2 regression model. Parameters measured consisted of serum lipid analytes: cholesterol, free cholesterol, phospholipids, triglycerides, apolipoproteins A1/A2/B100 and the B100/A1 ratio, and analyte distributions in different density classes of serum-lipoproteins: HDL, (density 1.063–1.210 kg/L), LDL (density 1.019–1.063 kg/L), intermediate-density lipoprotein (IDL, density 1.006–1.019 kg/L), and VLDL, 0.950–1.006 kg/L). The main lipoprotein classes HDL, LDL, VLDL were subdivided into different density sub-classes. A list of the 112-lipoprotein main and subfraction parameter annotations are provided in Supplementary Table S2. Four additional ratios representing a measure of low to high density particle distribution were calculated: i) the sum of larger LDL subfractions 1–3 (1.019–1.037 kg/L) to the sum of smaller LDL subfractions 4–6 (1.037–1.063 kg/L); (ii) LDL2 (1.031–1.034 kg/L) to LDL5 (1.040–1.044 kg/L); iii) sum of HDL subfractions 1–3 to HDL4, and (ii) the ratio of HDL1 to HDL4.

#### Statistical modelling and assessment of the lipoprotein data

All computation and visualization were performed using the statistical programming language and environment R (version 4.4.2), and mva.plots package (phenological/mva.plots) version 0.0.7. Parallel coordinate charts were generated to demonstrate the shift in lipoprotein distributions between the healthy-weight (BMI 18.5–24.99 kg/m²), overweight (BMI 25.0–29.99 kg/m²), and obesity (BMI ≥ 30 kg/m²) BMI categories. In the parallel coordinate plots the lipoprotein particle concentration levels (y-axis) were scaled from 0 to 1 to enable comparison across lipoprotein classes and subfractions. Participants with a BMI < 18.5 kg/m^2^ were excluded from this study.

The particle numbers for LDL and VLDL subfractions were normalised to the total particle number of their respective main lipoprotein classes (LDL and VLDL) for visualisation purposes only, to more accurately reflect the relative distribution of subfractions within each class. For all other analyses, subfraction concentrations were used in their original, untransformed form.

In the case of VLDL, phospholipid content was used as a surrogate for particle number. ApoB-100 can be used to calculate particle number assuming one molecule of apolipoprotein B100 per VLDL particle [20]. However, since the ApoB-100 value was only measured for total VLDL concentration but not for the subfractions VLDL1-5, total phospholipid concentration was used as a surrogate to estimate particle number. Although the phospholipid concentration is not fully equivalent to the ApoB-100 concentration, the correlation for total VLDL ApoB-100 (VLAB) with VLDL total phospholipid concentration (VLPL) is 0.976 and therefore deemed to be an adequate approximation (Supplementary Fig. 1A). Further confidence in this approximation can be gained from the correlation between LDL ApoB-100 (LDAB) with LDL total phospholipid concentration (LDPL) r = 0.968 (Supplementary Fig. 1B), which holds true for the LDL subfractions that gave corresponding correlation values between r = 0.980 and r = 0.995 (see Supplementary Fig. 1C–H). In the case of HDL, apolipoprotein-A1 (HDA1) was used to estimate particle number. The composition of free cholesterol (FC), phospholipids (PL), and triglycerides (TG) for each of the subfractions was part of the 112 lipoprotein parameters measured using the Bruker IVDr Lipoprotein Subclass Analysis (B.I.-LISA) method. Cholesterol ester (CE) was calculated by subtracting FC from total cholesterol (CH). FC, CE, PL, and TG were then combined to form a new total composition for each subfraction. Kruskal-Wallis rank sum tests were completed to analyse the average composition of lipoprotein particles, including CE, FC, PL, and TG, within the larger, more buoyant LDL (LDL1–LDL3) and the smaller, denser LDL particles (LDL4–LDL6).

Receiver Operating Characteristic (ROC) curve analysis was performed to evaluate the classification performance of selected lipoprotein ratios in discriminating between participants with healthy weight and participants with obesity. The area under the curve (AUC) was calculated to quantify discriminatory ability, with higher AUCs indicating better separation between outcome groups with values ranging from 0.5 (no discrimination) to 1.0 (perfect discrimination). AUCs were compared across candidate markers to identify those with the highest diagnostic utility. ROC analyses were conducted using the pROC package in R and smoothed ROC curves were generated using kernel-based estimators for visual clarity.

### Ultracentrifugation

To validate the results obtained from deconvolution of the NMR lipoprotein peaks, lipoprotein main fractions were isolated (from 6 participants in the healthy weight BMI category and 6 participants in the obesity BMI category) by sequential flotation ultracentrifugation based on established protocols using a Beckman Coulter Optima L-100 XP Ultracentrifuge equipped with a Beckman Type NVT-100 near vertical angle rotor. Using 1.5 mL of serum in a Beckmann OptiSeal tube (V = 4.9 mL), the main fractions were collected according to their desired density (VLDL d ≤ 1.006 g/mL, IDL d ≤ 1.019 g/mL, LDL d ≤ 1.063 g/mL, and HDL ≤ 1.210 g/mL) using NaBr gradients. For each, the upper lipoprotein fraction was taken, whereas the lower residue was density adjusted using NaBr to isolate the next fraction by centrifugation. The correct density slot of the isolated fraction was confirmed using a refractometer. The fractions were directly used for NMR measurements.

### NMR spectroscopic analysis of ultracentrifugation-separated lipoprotein fractions

For VLDL and IDL, 540 µL of the isolated fractions were mixed with 60 µL D_2_O containing 2 mM alanine for chemical shift adjustment using the midpoint of the CH_3_ doublet at 1.48 ppm and TSP. The solutions were transferred to 5 mm outer diameter NMR tubes and sealed. For LDL, and HDL, 160 µL of the isolated fractions were mixed with 40 µL of D_2_O containing 2 mM alanine and TSP. The solutions were transferred to 3 mm NMR tubes and sealed. All analyses were performed on 600 MHz Bruker Avance III HD spectrometer. For the main fractions VLDL, IDL, LDL, and HDL, a single one-dimensional (1D) NMR experiment was acquired at 310 K with solvent presaturation (128 scans, 98k data points, spectral width of 30 ppm, and an experiment time of 15 min).

## Results

### Study demographics

The cohort demographics (Supplementary Table 1) indicate some inherent differences between the BMI classified groups, specifically a lower participant age in the healthy weight group (BMI 18.5–24.99 kg/m^2^) compared to the overweight group (BMI 25–29.99 kg/m^2^) and the obesity BMI category (BMI ≥ 30 kg/m^2^) (median ages of 56.7 vs 57.9 vs 58.7 years, *p* < 0.001). Significantly elevated blood pressure (BP) levels were evident in the overweight and obesity BMI categories compared to the healthy weight group (median systolic/diastolic BP 121/72 mmHg vs 128/77 mmHg vs 138/81 mmHg, *p* < 0.001). Similarly, the CRP concentration was also significantly elevated in the overweight and obesity BMI categories compared to the healthy weight group (median CRP 0.9 vs 1.5 vs 2.8 mg/L, *p* < 0.001). A higher proportion of participants in the overweight and obesity BMI categories were taking lipid-lowering medication than in the healthy weight group (6.2% healthy weight vs 15% overweight vs 18% obesity BMI category, *p* < 0.001).

### Impact of BMI category on lipoprotein fractions

**Fig. 1:**
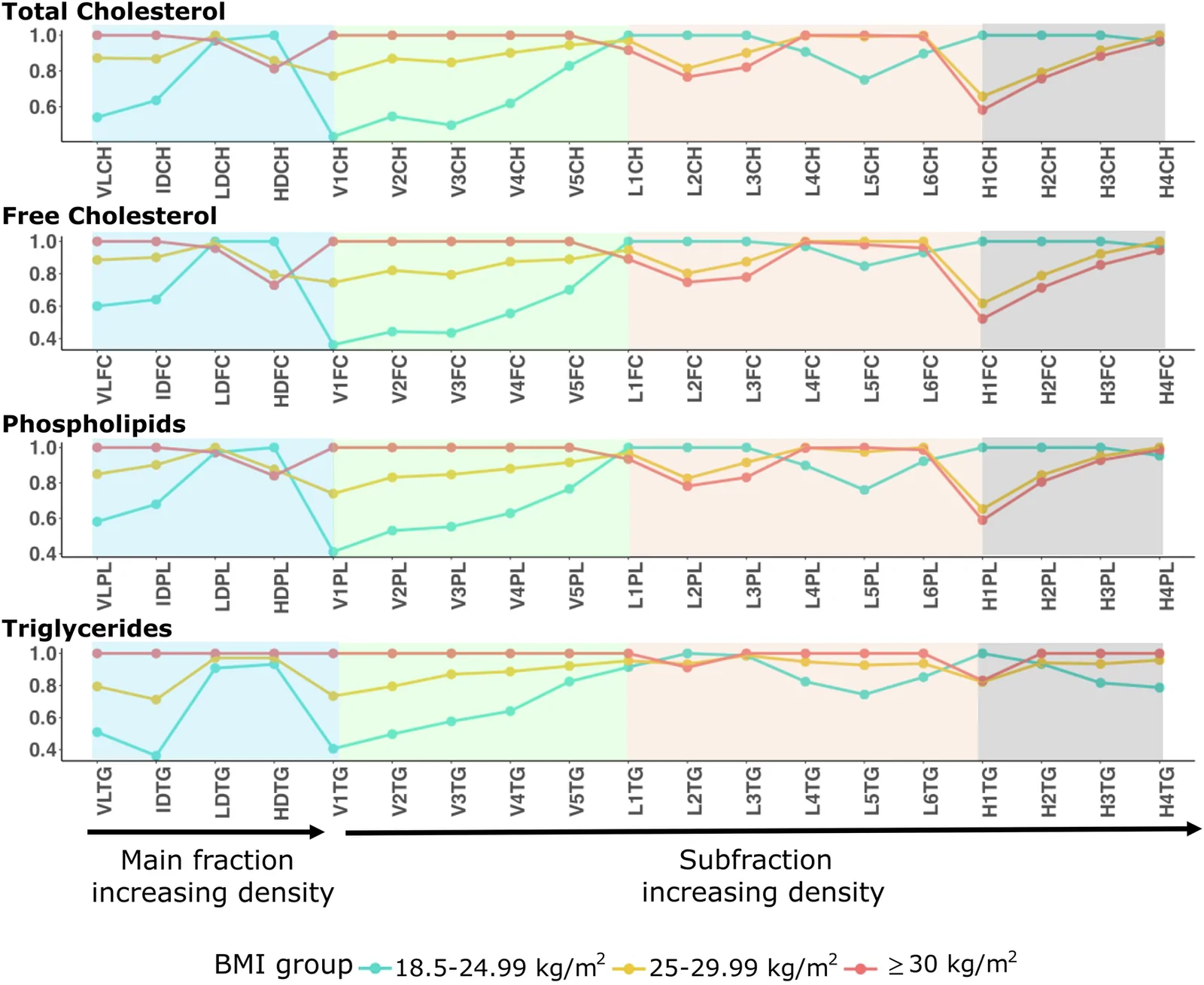
Parallel coordinate charts of Lipoprotein main fractions and subfractions stratified by BMI category. Healthy weight (green line, 18.5–24.99 kg/m^2^), overweight (yellow line, 25–29.99 kg/m^2^) and obese (red line, ≥30 kg/m^2^). Lipoprotein concentration (y-axis) is scaled (between 0 and 1) for each lipoprotein class to facilitate comparison of variables with different units or ranges. Background colour Blue: Main lipoprotein parameter, Green: VLDL, Red: LDL, Grey: HDL.

As illustrated in the scaled parallel coordinate plot (Fig. 1) participants in the healthy-weight BMI category demonstrated a more favourable lipoprotein profile compared with participants in the overweight and obesity BMI categories, with lower levels of VLDL (VLPN; healthy weight = 93 nmol/L, overweight = 138 nmol/L, obesity BMI category = 153 nmol/L) and reduced concentrations of CH, FC, TG and PL. IDL mirrored this effect while LDL had non-significant changes in CH, FC and PL and TG increased as BMI increased (LDTG; healthy weight = 17.0 mg/dL, overweight =18.1 mg/dL, obesity BMI categories = 18.7 mg/dL). For HDL, increasing BMI was associated with significantly lower CH, FC, and PL concentrations, while TG concentrations were directly correlated with BMI (Supplementary Table 6).

In addition to the higher total VLDL concentration observed in the obesity BMI category, differences in concentration were not uniform across the VLDL density range. As illustrated in the parallel plot, the lowest-density subfraction (VLDL-1) showed the greatest difference between the healthy weight, overweight, and obesity BMI categories. The healthy weight group had the lowest VLDL1 CH, FC, PL and TG concentrations, the overweight group intermediate concentrations, and the obesity BMI category had the highest concentrations, with these differences between BMI categories narrowing for the higher-density VLDL subfractions. For the LDL subfractions clear differences were observed between the sum of LDL1–3 and LDL4–6. Reductions in CH, FC and PL were observed as BMI increased for subfractions LDL1–3, while CH, FC, and PL increased as BMI increased for subfractions 4–6. All the LDL subfraction TG concentrations were significantly different between the BMI groups except LDL subfraction 3. Lower CH, FC, PL and TG were observed in HDL subfraction 1 with increases in BMI, while CH, FC, PL and TG were lower in HDL subfraction 2 as BMI increased. HDL subfraction 3 showed a similar pattern to HDL subfraction 2 but an increase in TG was observed as BMI increased (H3TG; healthy weight = 1.87 mg/dL, overweight = 2.13 mg/dL, obesity BMI category = 2.26 mg/dL). HDL subfraction 4 FC was not significantly different across the BMI groups, however CH, FC and PL were higher in the overweight and obesity BMI categories compared to the healthy weight group (all concentrations and significance can be found in Supplementary Table 3).

### Associations between BMI category and the distribution of lipoprotein subfractions were observed for particle number, but not for the composition of lipoprotein particles

**Fig. 2:**
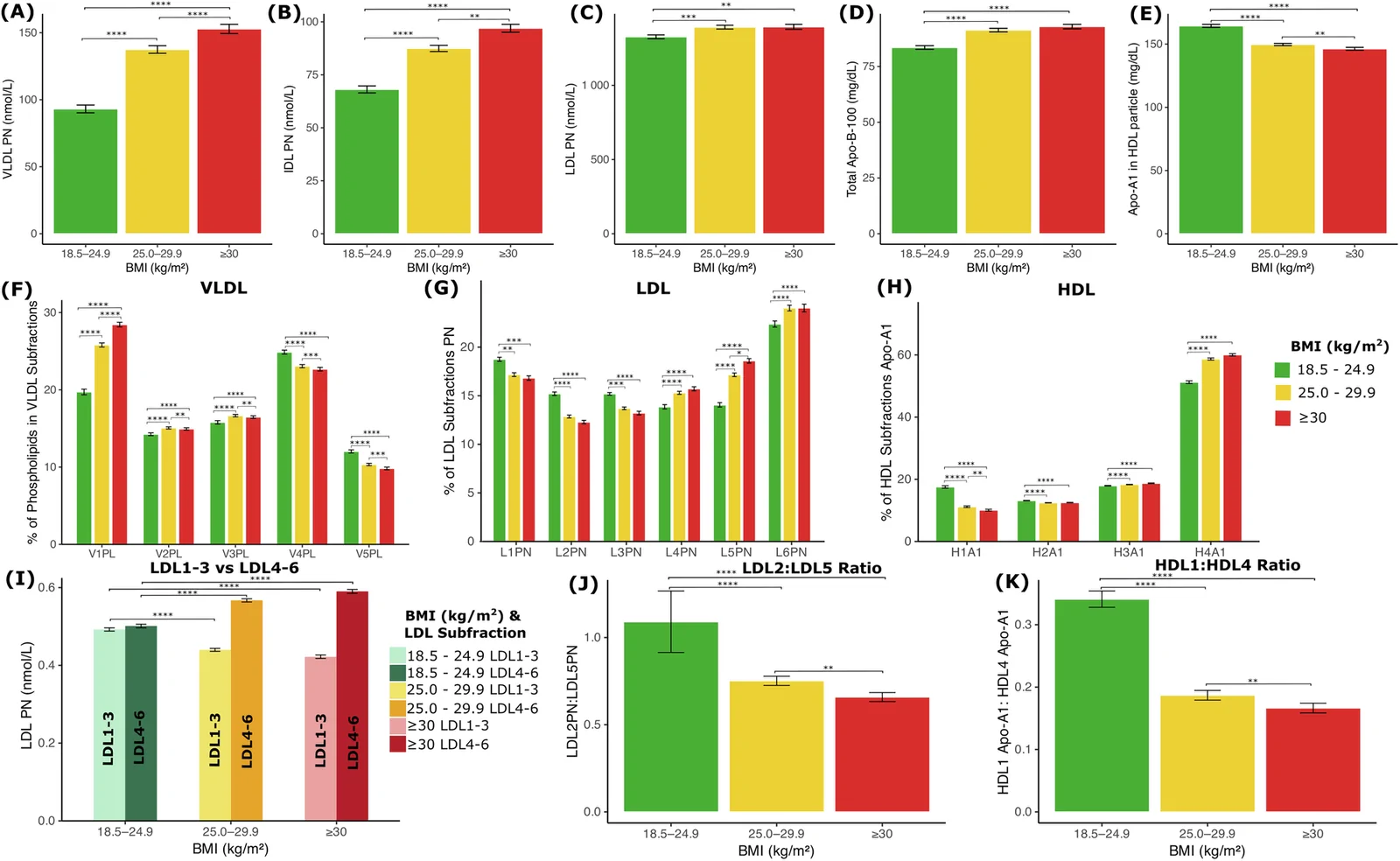
Bar plots showing the lipoprotein particle number distributions for the main fractions, subfractions and ratios. **A** VLDL, **B** IDL, **C** LDL, **D** total ApoB100 and **E** HDL across BMI categories: healthy weight (18.5–24.99 kg/m^2^), overweight (25–29.99 kg/m^2^) and obesity BMI category (≥30 kg/m^2^). **F**–**H** BMI associated differences in particle number distributions for VLDL, LDL and HDL subfractions, respectively. **I** BMI associated differences in particle number distribution ratio for the sum of LDL1–LDL3 vs. LDL4–LDL6. **J** BMI associated differences in the particle number ratio of particle number for LDL2:LDL5. **K** BMI induced differences in the ratio of particle number for HDL1:HDL4 using ApoA1 subfraction concentrations. Only significant differences between BMI categories are highlighted according to *p*-value (*p < 0.05; **p < 0.01; ***p < 0.001; ****p < 0.0001).

To determine whether the observed differences in lipoprotein subfraction concentrations were driven by changes in particle number or by alterations in particle composition, particle numbers were compared between the healthy weight, overweight and obesity BMI categories across the main lipoprotein classes and their subfractions. As BMI increased total VLDL, IDL (IDPN; healthy weight = 68 nmol/L, overweight = 87 nmol/L, obesity BMI category = 97 nmol/L) and LDL (LDPN; healthy weight = 1327 nmol/L, overweight = 1392 nmol/L, obesity BMI category = 1394 nmol/L) increased, while HDL, estimated using apo-A1 concentration as a proxy, reduced (HDA1; healthy weight = 164 mg/dL, overweight = 150 mg/dL, obesity BMI category = 146 mg/dL) (Fig. 2A–E).

Subfraction particle number changes were observed across VLDL, LDL and IDL as BMI increased. VLDL subfractions (calculated using phospholipid as proxy, V1PL–V5PL) 1–3 were significantly higher with increasing BMI, while subfractions 4–6 significantly lowered. Total VLDL-PL (VLPL) concentration was elevated in the obesity BMI category (22 mg/dL) in comparison to the overweight (19 mg/dL) and healthy weight (13 mg/dL) groups, with the largest contribution from VLDL-1, while the relative proportion of VLDL-5 was reduced compared to the healthy weight group (Fig. 2F).

Although total LDL concentration did not differ significantly between the overweight and obesity BMI categories (Fig. 2C, Supplementary Table 3), the relative concentration of small, dense LDL particles increased with BMI (LDL4–6; 1.037–1.063 kg/L) (Fig. 2G), while the larger less dense LDL particles reduced (LDL1-3; 1.019–1.037 kg/L). This was evident across all LDL-associated lipids, including FC, CE, PL, and, to a lesser extent, TG as shown by the scaled median concentrations in Fig. 1, prompting the evaluation of large to small lipoprotein subfraction ratios. For the healthy weight group, no significant difference was observed between the large and small LDL subfractions, while the overweight and obesity BMI categories demonstrated higher proportions of small, dense LDL particles (Fig. 2I, J).

For HDL, the largest subfraction (HDL1 (H1A1); 1.063–1.100 kg/L) was significantly lower in participants in the obesity BMI category compared to those in the healthy weight group (healthy weight = 29 mg/dL, overweight = 17 mg/dL, obesity BMI category = 14 mg/dL). In contrast, the smallest subfraction (HDL4 (H4A1); 1.125–1.210 kg/L) showed little variation between the overweight and obesity BMI categories (healthy weight = 82 mg/dL, overweight = 87 mg/dL, obesity BMI category = 86 mg/dL) (Fig. 2H).

The differential impact of BMI across the density spectrum of lipoprotein subfractions suggests that variations in particle number drove the observed differences in lipoprotein concentrations (Supplementary Figs. 2–4, Supplementary Tables 4-5). To support these findings, ultracentrifugation was performed on plasma from six participants in the healthy weight BMI category and six participants in the obesity BMI category (Supplementary Fig. 5) to isolate LDL fractions. Spectral analysis of these isolated LDL fractions revealed a shift toward lower resonance frequencies in samples from participants in the obesity BMI category, indicating a higher proportion of small, dense LDL particles (L4–6PN), consistent with the B.I.-LISA result.

### Effect of statin use on lipoprotein profiles

The cohort was stratified by statin use (*n* = 1563 no statin use; *n* = 243 taking statin medication) and both subsets were re-analysed independently. For both the healthy weight (Supplementary Fig. 6) and obesity BMI categories (Supplementary Fig. 7) lower total LDL particle number (18% reduction in the healthy BMI category, *p*-value = 1.89 × 10^−5^, 22% reduction in the obesity BMI category, *p*-value = 2.53 × 10^−16^) while no significant differences were observed for total VLDL, IDL and HDL (Supplementary Figs. 6A, 7A). For both the healthy weight and obesity BMI categories, significant differences were observed in the LDL subfractions but not VLDL or HDL. In the healthy weight group, particle numbers of LDL subfractions LDL1–LDL4 were lower (Supplementary Table 6) while all LDL subfractions in the obesity BMI category were lower (Supplementary Table 7). In the obesity BMI category, a significant difference was observed between the larger LDL subfractions (LDL1–LDL3) and smaller LDL subfractions (LDL4–6) (Supplementary Fig. 7B). There was no significant difference between the large and small subfractions in the healthy weight group indicating the change in LDL particle sizes occurs regardless of statin use. (Supplementary Fig. 6B).

Lower total LDL particle numbers were also accompanied by compositional changes. Statin use significantly lowered CE and PL and TG were higher in the larger less dense LDL both in the healthy weight and obesity BMI categories. While in the smaller, denser LDL, CE (healthy weight BMI category *p*-value = 4.00 × 10^−3^, obesity BMI category *p*-value = 2.94 × 10^−17^) was significantly lower and triglycerides were significantly higher (healthy weight BMI category *p*-value = 8.00 × 10^−3^, obesity BMI category *p*-value = 3.08 × 10^−11^) with statin use. Interestingly, FC did not change, and PL was significantly higher only in the obesity BMI category (obese *p*-value = 1.18 × 10^−8^) (Supplementary Tables 8, 9).

### Assessment of the association of lipoprotein subfraction ratios with BMI

**Fig. 3:**
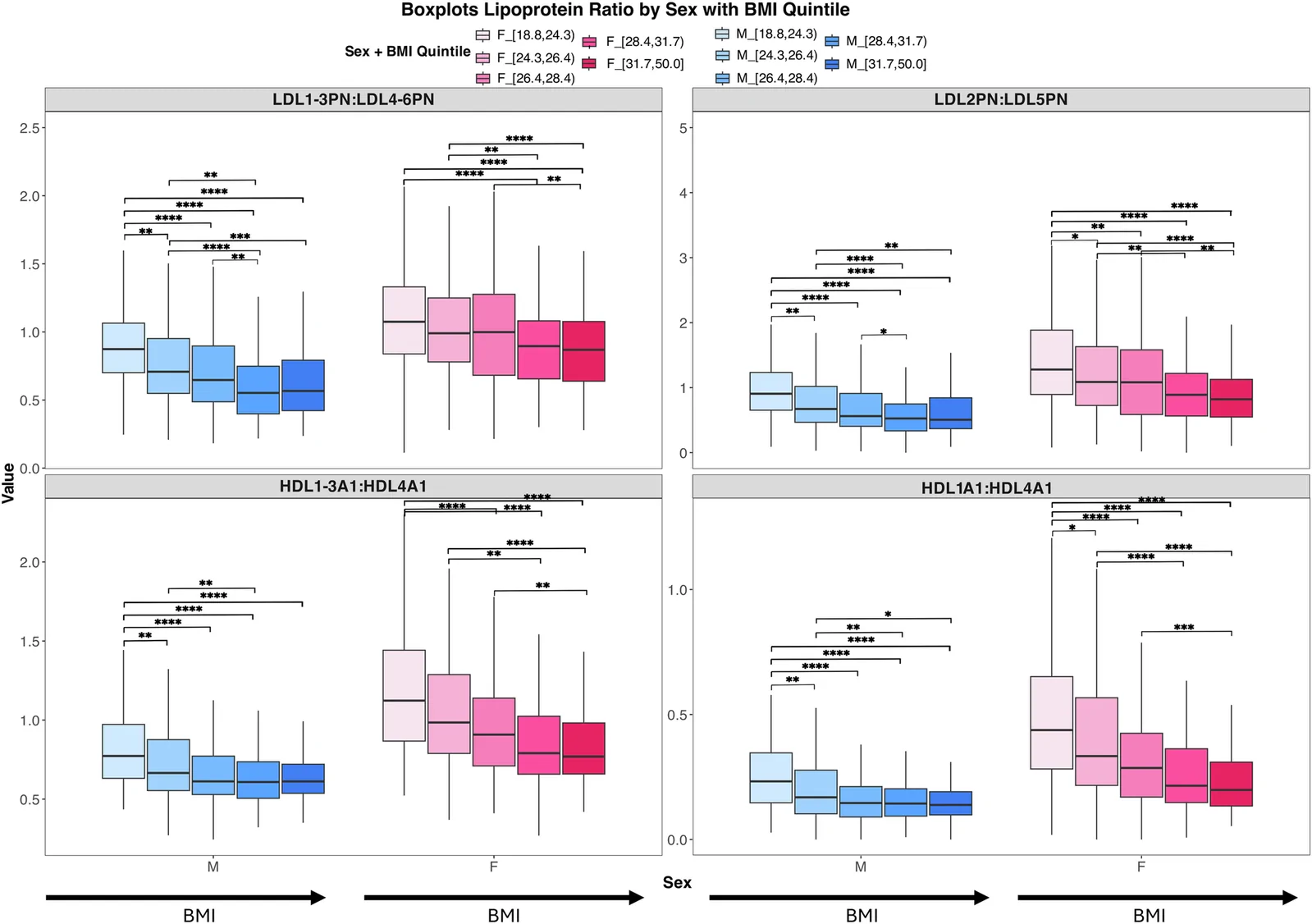
Distribution of lipoprotein ratios by sex across BMI Quintiles. LDL1–3PN:LDL4–6PN: the ratio of the sum of LDL 1–3 to the sum of LDL 4–6 particle numbers. LDL2PN:LDL5PN: the ratio of LDL 2 to LDL 5 particle numbers. HDL1-3A1:HDL4A1: the ratio of the sum of HDL 1–3 to HDL4 particle numbers. HDL1A1:HDL4A1: the ratio of HDL1 to HDL4 particle numbers. Only significant differences between BMI categories are highlighted with *p*-value (*p < 0.05; **p < 0.01; ***p < 0.001; ****p < 0.0001).

The relationship between BMI and LDL particle distribution across density subfractions was further explored using two particle number ratios: (i) the ratio of the sum of larger LDL subfractions 1–3 (1.019–1.037 kg/L) to the sum of smaller LDL subfractions 4–6 (1.037–1.063 kg/L), and (ii) the ratio of LDL2 (1.031–1.034 kg/L) to LDL5 (1.040–1.044 kg/L). These ratios were stratified by sex and compared across the BMI quintile ranges (Fig. 3).

**Fig. 4:**
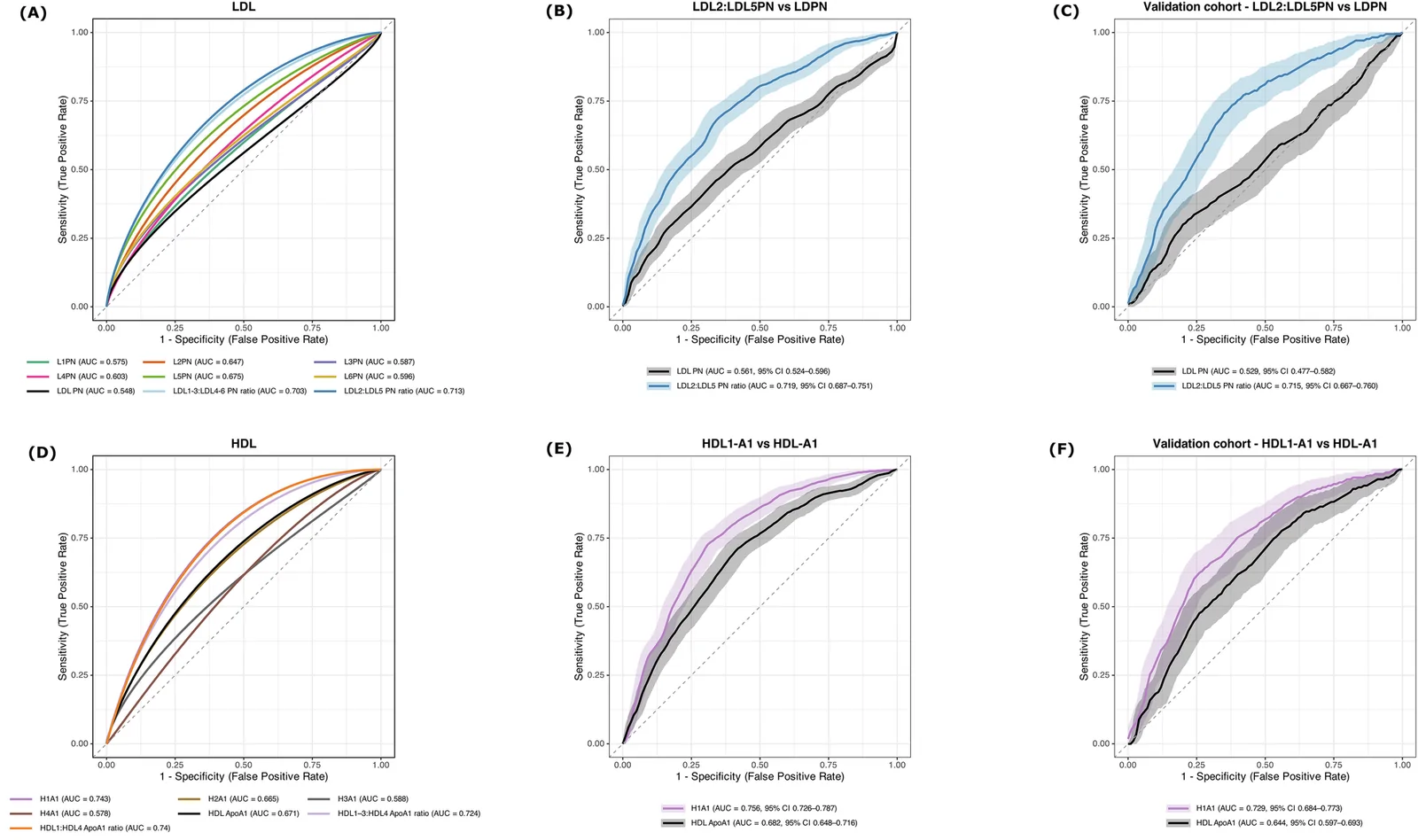
Area under the Receiver Operator Curves (AUROC) for each LDL and HDL main fractions, subfractions and ratios when modelling the healthy weight groups versus the obese group. **A** The LDL particle number ratios and subfractions, **B** LDL2:LDL5 particle number ratios vs. total LDL particle number, **C** LDL2:LDL5 particle number ratios vs. total LDL particle number for the validation cohort, **D** the HDL apolipoprotein A1 ratios and subfractions, **E** HDL 1:HDL4 apolipoprotein A1 ratios vs. total HDL apolipoprotein A1, and **F** HDL 1:HDL4 apolipoprotein A1 ratios vs. total HDL apolipoprotein A1 for the validation cohort.

While the concentrations for some lipoprotein fractions differed between men and women, the box-and-whisker plots, stratified by sex and BMI quintile, demonstrated a progressive decline in both ratios with increasing BMI, indicating a shift from larger, more buoyant LDL particles to smaller, denser subfractions in participants with higher BMI (Fig. 3A–C). Both LDL particle number ratios showed good discriminatory ability in differentiating the healthy weight and obesity BMI categories. The area under the receiver operating characteristic curve (AUROC) was 0.71 for the LDL2:LDL5 ratio and 0.70 for the LDL1–3:LDL4–6 ratio, both outperforming the discriminative capacity of total LDL and individual LDL subfractions, whose AUROCs ranged from 0.55 (LDLPN) to 0.68 (LDL5) (Fig. 4A), Full statistical details, including significance values for each subfraction and ratio, are provided in Supplementary Table 10. The LDL2:LDL5 ratio for the validation cohort also outperformed LDL PN with similar AUROCs observed (LDL2:LDL5 AUC = 0.72, LDL PN AUC = 0.53) (Fig. 4C).

Similarly, two ratios were calculated for HDL particle numbers: (i) the ratio of the sum of HDL subfractions 1–3 to HDL4, and (ii) the ratio of HDL1 to HDL4 (Fig. 3D–F). Both ratios exhibited a progressive shift with increasing BMI, reflecting a relative reduction in larger HDL particles and an increase in smaller, denser HDL particles. When comparing healthy weight and obesity BMI categories, HDL1 apolipoprotein A1 alone (AUROC = 0.74) demonstrated equal or superior discriminatory performance compared to either ratio (HDL1:HDL4 ratio (AUROC = 0.74); HDL1–3:HDL4 ratio (AUROC = 0.72)) whereas total HDL apo-A1 only generated an AUROC of 0.67. HDL1 apolipoprotein A1 was also shown to have superior discriminatory performance compared to total HDL in the validation cohort (Fig. 4F). To determine whether the ratios remained significant in the presence of comorbidities, participants without cardiovascular disease were compared with participants with cardiovascular disease (Supplementary Fig. 8). It was found that all ratios remained significant between the healthy weight and obesity BMI categories. In a recent study, Conde et al. investigated lipoprotein subfraction changes in relation to comorbidities (diabetes and hypertension) and reported a similar shift to that observed in this study across BMI categories, suggesting these changes could be associated with chronic inflammation [21].

## Discussion

### The impact of obesity on the lipoprotein profile

The lipoprotein profiles measured in the present study were consistent with established dyslipidaemic patterns associated with obesity characterised by elevated concentrations of VLDL, IDL, and small, dense LDL particles, accompanied by reduced HDL concentration. This atherogenic profile is well documented to contribute to the increased cardiovascular risk observed in people with obesity [4, 22]. The distribution of LDL subfractions within the main class differed, with higher concentrations of the smaller, denser, and more atherogenic LDL4–LDL6 particles in the overweight and obesity BMI categories. This redistribution towards small, dense LDL subfractions is clinically relevant given their established association with increased cardiovascular risk [23].

The characteristic alterations in LDL and HDL profiles observed in obesity are primarily driven by triglyceride-mediated qualitative changes in apoB-100 containing and in apoA-containing lipoproteins, respectively [4, 24]. The alterations in LDL and HDL profiles are also observed in people with diabetes [2, 25]. In the context of insulin resistance and elevated circulating apoC-III, hepatic overproduction of VLDL particles, each containing a single molecule of apoB-100, is accompanied by delayed peripheral catabolism, leading to an expanded pool of large, triglyceride-rich VLDL. This excess promotes enhanced lipid exchange via cholesteryl ester transfer protein (CETP), enriching LDL and HDL particles with triglycerides. Triglyceride-enriched LDL and HDL are subsequently hydrolysed by hepatic lipase, whose activity is increased in insulin-resistant states, producing increased numbers of small, dense LDL and HDL particles as observed in this study in participants across BMI categories. The increase in small, dense LDL particles, alongside the overall elevated number of apoB-100-containing lipoproteins, contributes to heightened atherogenic risk. Concurrently, increased free fatty acid flux from insulin-resistant visceral adipose tissue drives hepatic lipogenesis, further exacerbating VLDL overproduction and promoting hepatic steatosis.

In terms of particle number, our findings for VLDL (over 50% higher in participants in the obesity BMI category) and HDL (~10% lower in the obesity BMI category) align with those reported in an American cohort [26]. However, in contrast to their observation of a 100% increase in both LDL and IDL particle numbers in participants with obesity, we detected a more modest 43% increase in IDL and a 5% increase in total LDL (Fig. 2). This is consistent with other studies, which have reported either weak or non-significant correlations between BMI and LDL concentrations [27]. There was a highly significant shift in the distribution of LDL subfractions. Specifically, participants in the obesity BMI category exhibited a redistribution from larger, more buoyant LDL subfractions (LDL1–3: 1.019–1.037 kg/L) to smaller, denser subfractions (LDL4–6: 1.037–1.063 kg/L). This shift was driven by changes in particle number, as confirmed by ^1^H NMR spectral profiles of LDL fractions isolated by ultracentrifugation from serum samples of participants in the healthy weight and obesity BMI categories. A similar pattern was also observed within the HDL lipoprotein classes, with a progressive shift from larger to smaller, denser HDL subfractions in association with increasing BMI.

### Obesity induces a shift in LDL lipoprotein subfractions towards a more atherogenic profile

The observed shift in LDL subfraction distribution from larger, less dense particles (LDL1–3) to smaller, denser fractions (LDL4–6) aligns with previous findings reported using density ultracentrifugation [28] and NMR spectroscopy [29]. In the present study, participants in the healthy weight BMI category exhibited approximately equal concentrations of the larger (1.019–1.037 kg/L) and smaller (1.037–1.063 kg/L) LDL subfractions. However, in the obesity BMI category, this balance shifted significantly toward a greater proportion of the smaller, denser LDL subfractions (Fig. 2I). Importantly, this was not influenced by statin use, as stratification by statin intake showed no significant impact on LDL particle number proportions but did reduce LDL particle number concentrations and alter composition (Supplementary Figs. 6, 7, Supplementary Tables 6–9). The advantage of using the ratio for LDL2:LDL5 particle number (AUC 0.72) over the total LDL (AUC 0.56) or any of the LDL sub-fractions alone is apparent from the AUROC values (Fig. 4), with the ratio outperforming the other parameters in discriminating between BMI categories. The ratio also outperformed the total apolipoprotein-B-100 concentration (Supplementary Fig. 9) in terms of characterising obesity, where the AUC was 0.61.

Stratification of the main lipoproteins into subfractions can deliver more nuanced information on the relationship between BMI, lipoprotein profiles, and cardiovascular disease risk. Smaller denser LDL (LDL4–LDL6) particles have a lower affinity for the LDL receptor, causing them to remain in circulation longer and they are more susceptible to oxidative modification and glycation, as well as potentially interacting more easily with proteoglycans of the arterial wall; all contributing to their increased atherogenic potential [30]. It has been estimated that people with elevated levels of small, dense LDL may have up to a seven-fold higher risk of developing coronary heart disease [23]. Additionally, intra-abdominal adiposity has been positively correlated with the presence of smaller dense LDL subfractions [31]. Supporting these findings, another study reported that higher BMI was directly associated with increased concentrations of the smallest LDL subfractions (LDL5 and LDL6), accompanied by a reduction in medium-density LDL particles (LDL2 and LDL3) [32]. Consistent with these observations, several dietary and exercise intervention studies have demonstrated beneficial shifts in LDL subfraction profiles, with an increase in larger, less dense LDL (LDL1–3; <1.04 g/mL) particles following lifestyle modification [33], with bariatric surgery inducing a reduction in triglyceride-rich lipoproteins [34]. Accumulating evidence indicates that the total number of LDL particles is a stronger determinant of ASCVD risk [35]. Supporting this, recent large-scale data from the UK Biobank demonstrate that the total concentration of apoB-100 containing particles, rather than their size or compositional differences, is the most robust predictor of ASCVD events [36].

The B.I.LISA NMR method for calculating the main and subfractions of lipoproteins generates six subfractions for LDL [11]. Other studies report anywhere between 3 and 7 LDL density substrata citing reference ranges in either nm or g/ml using a range of methods including ultracentrifugation, gradient gel electrophoresis, iodixanol density gradient and high-performance liquid chromatography [37–39]. Despite the different analytical methods and LDL sub-categories, there is widespread agreement that small dense LDL is associated with adverse cardiometabolic impact.

### Differential relationship of large and small high-density lipoprotein subfractions with obesity

In our study, higher BMI was associated with a significantly lower particle number for HDL1 and HDL2 (*p* < 0.001) (Fig. 2H and Supplementary Table 3), as approximated from the measured ApoA1 concentrations. Low concentrations of large HDL particles have been shown to be associated with impaired metabolic health. Notably, it is well established that large and small HDL subfractions exhibit distinct biological behaviours [40], a finding supported in this study. While HDL1 and HDL2 levels were reduced in participants in higher BMI categories, the concentration of the smallest size subfraction, HDL4 increased. These findings are consistent with the work of Sergi et al., who demonstrated that increased visceral adipose tissue, elevated circulating triglycerides, and insulin resistance were associated with a redistribution of cholesterol into small, dense HDL particles [41]. Accordingly, several diet studies show that weight loss is frequently accompanied by an increase in the proportion of large HDL particles [42], suggesting that dynamic changes in HDL subfraction distribution may reflect metabolic improvements rather than merely static lipid levels.

We previously demonstrated that SPC_1_, an NMR-derived marker of inflammation which yields information on the phospholipid content in HDL4 significantly increased as BMI increased whereas SPC_2_, derived from the phospholipid content within HDL subfractions 1–3 was inversely correlated with BMI [43]. The findings of this study support previous work which used an independent NMR spectroscopic method to measure the lipoprotein parameters. Low HDL concentrations in people with obesity have been associated with enhanced uptake of HDL1–3 by adipocytes and additionally a decrease in the conversion of pre-beta1 to pre-beta2 particles (which overlap in size with small HDL but cannot be directly measured using NMR) suggesting impaired recovery of cholesterol and reverse cholesterol transport involving the transfer of FC and PL to nascent HDL particles via the ATP-binding cassette transporter A1 (ABCA1) [44]. The observed decrease in large HDL formation because of impaired reverse cholesterol transport arises from inflammation driven increases in cholesteryl ester transfer protein (CETP) activity and decreased lecithin-cholesterol acetyltransferase (LCAT) activity. This process normally serves to clear excess cholesterol from tissues including the artery walls and reduces the formation of macrophage foam cells. The FC in HDL particles is subsequently esterified into cholesterol esters by LCAT, which move to the hydrophobic core of HDL allowing the chain of formation from nascent to small, then large HDL for excretion both directly and via bile. This pattern can be seen clearly in the current study whereby HDL4 is inversely correlated with HDL1 and HDL2 (Fig. 2). Indeed, it has been shown that diminished mean HDL size correlates with increased cardiovascular disease in large scale clinical studies [45].

Although HDL cholesterol is typically considered to be “good cholesterol”, recent research indicates that HDL can behave similarly to LDL in terms of cardiovascular disease risk [46]. An increased number of small-sized HDL particles has been associated with a higher risk of incident ASCVD, particularly in women; however, this association appears to be attenuated after adjusting for total LDL particle number [47]. Ganjali et al. discussed the U-shaped association in plasma HDL cholesterol concentrations in atherosclerotic cardiovascular disease (ASCVD) mortality based on multiple epidemiology studies [48]. This challenges the current use of total HDL cholesterol as a reliable indicator of cardiovascular health, highlighting the need for an extended lipoprotein profile that can provide more reliable associations with metabolic health.

In people with obesity, the generation of small, dense LDL and HDL particles is largely driven by triglyceride enrichment and subsequent lipolysis via hepatic lipase, a process exacerbated by insulin resistance [4]. Importantly, it is now well established that the total number of atherogenic apoB-100–containing lipoproteins, which include VLDL, IDL, and LDL particles, is a primary determinant of ASCVD risk, irrespective of particle size. Recent studies further highlight the role of triglyceride content within these particles, with both LDL TG and HDL TG emerging as independent predictors of ASCVD events [49], reinforcing the importance of assessing both lipoprotein number and composition in dyslipidaemia associated with higher BMI.

We have shown the ratio of the particle number in small to large LDL subfractions is more strongly associated with higher BMI categories than total LDL, individual LDL subfractions or ApoB-100. For HDL, the HDL1 subfraction performed comparably to the ratio for large (HDL1–3) to small (HDL4) subfractions, which suggests a reduction in HDL1 is the key parameter defining the HDL signature associated with obesity.

### Limitations and future direction

Our study has several limitations that should be acknowledged. The NMR-derived parameters do not include apoC-III, which has been proposed as a major determinant of hypertriglyceridemia and has been associated with compositional changes in LDL and HDL [4]. Fasted plasma samples were collected for both populations and therefore the applicability to non-fasted samples has not been determined. However, a study by Langlois et al. found that fasting did not have a substantial effect on LDL particle numbers [50]. When considering the LDL and HDL particle number ratios there were sex differences in HDL with women demonstrating a higher small to large ratio across all BMI quintiles. Another important consideration is the accessibility of our analytical approach. High-resolution spectroscopy, while highly informative, requires specialised equipment such as ¹H-NMR 600 MHz spectrometers, that are currently not widely available in clinical settings. This limits the immediate clinical translation of our findings and highlights the need for smaller, cost-effective platforms that can facilitate broader implementation in routine practice such as the clinically accessible benchtop NMR [51, 52]. As with all proprietary software, the lack of transparent validation of the initial model can be problematic, as highlighted by Krauss et al. [53], nevertheless the ultracentrifugation of LDL subfractions 1–6 showed consistency with the calculated concentrations from the NMR spectra.

Statistically, the normalization of the particle numbers for LDL and VLDL subfractions to the total particle number of the main LDL and VLDL parameters rather than using absolute concentrations can introduce bias since the ability to detect whether total particle number itself differs between groups is lost. In the case of VLDL, phospholipid content was used as a surrogate for particle number. Using phospholipid content as a surrogate for VLDL particle number assumes a consistent phospholipid content per particle across individuals and subfractions, which may not hold true in states such as insulin resistance where lipoprotein composition is altered. Thus, if VLDL particles in higher BMI categories are more triglyceride-rich and phospholipid-poor, this assumption could misrepresent true particle number, leading to systematic bias in estimating subfraction distributions and their associations with disease outcomes.

While ApoA-1 was used as a surrogate for HDL particle number, it is known that there is an increase in ApoA-1 as particle size increases. However, ApoA-I is an obligate structural component of HDL and because the number of ApoA-I molecules per HDL particle varies within a relatively narrow range under physiologic conditions, circulating ApoA-I concentration can serve as an approximate surrogate of HDL particle number, particularly when direct particle measurements are unavailable. Furthermore, the effects of weight loss interventions on the lipoprotein profiles, and their relationship with ASCVD outcomes, warrant further investigation. Addressing these limitations in future studies will be essential for refining and expanding the clinical utility of this approach. Interventional studies targeting triglyceride metabolism, such as those involving apoC-III, ANGPTL3, and ANGPTL4 inhibitors, will be particularly important to determine whether hypertriglyceridemia is the causal driver of changes in lipoprotein particle concentrations.

## Conclusions

The NMR generated lipoprotein panel allows a more nuanced profile of BMI-associated dyslipidaemia to be obtained over the conventional assays that are offered by clinical pathology laboratories. If constrained to a single lipoprotein parameter for the metabolic characterization of higher BMI, apoB-100 provides a robust and clinically informative marker, capturing the atherogenic lipoprotein burden with established prognostic relevance. However, the addition of an NMR-derived lipoprotein panel, encompassing particle number and subfraction distribution, offers a more nuanced and comprehensive profile of lipid metabolism, enabling deeper insights into lipoprotein alterations with obesity and their cardiometabolic consequences. Given that the panel of NMR parameters can be generated rapidly and in high throughput (4 mins per sample), we propose that additional lipoprotein parameter ratios be considered in assessing lipid dysregulation associated with higher BMI. We have shown that the ratio of large to small LDL cholesterol is significantly decreased in the obesity BMI category and that this change is largely, although not entirely, driven by an increase in the number of small LDL particles. Therefore, we propose that using the LDL2:LDL5 ratio provides a robust metric since these parameters sit in the middle of the range and are measured with greater accuracy than for example LDL6. Furthermore, the reduction in large HDL particles in the obesity BMI category may be more informative than consideration of total HDL cholesterol concentration. The superior AUROC of the lipoprotein-based model relative to conventional obesity metrics suggests that it captures metabolic alterations associated with higher BMI that are not adequately reflected by BMI alone, supporting its potential utility as a more sensitive marker of BMI-associated metabolic dysfunction. A key unresolved question, requiring confirmation in prospective studies, is whether total apoB-100 containing particle number remains a superior predictor of ASCVD outcomes compared with the concentration of specific lipoprotein subclasses, a distinction highlighted in recent data [36].

In summary, we have characterised the lipoprotein phenotype associated with obesity and demonstrated that NMR-based lipoprotein profiling can be expanded to capture more subtle differences in LDL and HDL subfraction distributions than are currently available through routine clinical testing, providing greater insight into cardiometabolic risk assessment.

## Supplementary information

Supplementary Information
