# Supplementary material for: High resolution lipoprotein subfraction profiling identifies obesity associated distributional changes in LDL and HDL subfractions and particle ratios
Source: Int J Obes (Lond). 2026 Aug 13;50(10):2195–204. doi: 10.1038/s41366-026-02172-6 (PMC13722595; doi:10.1038/s41366-026-02172-6)
Supplement: Supplementary file 1 [file 41366_2026_2172_MOESM1_ESM.pdf]

High Resolution Lipoprotein Subfraction Profiling Identifies Obesity Associated Distributional Changes in LDL and HDL Subfractions and Particle Ratios

Novia Minaee[1], Reika Masuda[1], Philipp Nitschke[1], Drew Hall[1], Jennie Hui[2,3], Michael L Hunter[2,4], Leon Flicker[5], Graeme J Hankey[5,6], Osvaldo P Almeida[5,7,8], Dick C Chan[5], Gerald F Watts[5,9], Muhammad Saeed Ahmad[10,11], Huda M. Alkreathy[11.,12], Zoheir Abdullah Dhamanouri[11,12], Hartmut Schäfer[13], Manfred Spraul[13], Claire Cannet[13], John P Beilby[2,14], Bu B Yeap[5,15], Oscar Millet[16], Julien Wist[1,17], Jeremy K Nicholson[18,20], Samantha Lodge[1], Elaine Holmes[19,20].

[1]*Centre for Computational and Systems Medicine, Health Futures Institute, Murdoch University, Harry Perkins Building, Perth, WA6150, Australia.*

[2]*Busselton Population Medical Research Institute, Busselton, WA6280, Australia.*

[3]*Department of Diagnostic Genomics, PathWest Laboratory Medicine, Queen Elizabeth II Medical Centre, Perth, WA6009, Australia.*

[4]*School of Population and Global Health, University of Western Australia, Perth, WA6009, Australia.*

[5]*Medical School, University of Western Australia, Perth, WA6009, Australia.*

[6]*Perron Institute for Neurological and Translational Science, Western Australia, 6009, Australia.*

[7]*Institute for Health Research, University of Notre Dame Australia, Fremantle, WA6959, Australia; Medical School, University of Western Australia, Perth, WA6009, Australia.*

[8]*School of Population Health, Curtin University, Perth, WA 6102, Australia.*

[9]*Department of Cardiology and Internal Medicine, Royal Perth Hospital, WA6000, Perth, Australia.*

[10]*Department of Veterinary Medicine, University of Cambridge, Cambridge, CB3 OES, UK.*

[11]*Drug Metabolism Unit, King Fahad Medical Research Center, King Abdulaziz University, Jeddah 21589, Saudi Arabia.*

[12]*Department of Clinical Pharmacology, Faculty of Medicine, King Abdulaziz, Jeddah 21589, Saudi Arabia.*

[13]*Bruker Biospin GmbH & Co. Kg, Rudolf-Plank Straße 23, 76275 Ettlingen, Germany.*

[14]*School of Biomedical Sciences, University of Western Australia, Perth, WA6009, Australia.*

[15]*Department of Endocrinology and Diabetes, Fiona Stanley Hospital, Perth, WA6150, Australia.*

[16]*Precision Medicine and Metabolism Laboratory, CIC bioGUNE, Parque Tecnológico de Bizkaia, Bld. 800, 48160, Derio, Spain*

[17]*Chemistry Department, Universidad del Valle, Cali 76001, Colombia.*

[18]*Institute of Global Health Innovation, Imperial College London, London, SW7 2AZ, UK.*

[19]*Department of Metabolism, Digestion and Reproduction, Faculty of Medicine, Imperial College London, London, SW7 2AZ, UK.*

[20]*School of Medicine, The Hong Kong University of Science and Technology, Hong Kong, China.*

KEYWORDS:
Obesity, lipoprotein, VLDL, LDL, HDL, Metabolic profiling, Metabolomic, Metabonomic, NMR spectroscopy, cardiovascular risk.

CONTACT INFO:

Dr. Samantha Lodge
Centre for Computational and Systems Medicine
Health Futures Institute
Murdoch University WA 6150
Australia
sam.lodge@murdoch.edu

Professor Elaine Holmes
School of Medicine
The Hong Kong University of Science and Technology
Clear Water Bay
Kowloon
Hong Kong
China
elaineholmes@ust.hk

Table S1. **Demographics of the Australian cohort (N = 1806) showing median ± standard deviation**

Group	Healthy weight	Overweight	Obese	
**BMI**	**BMI 18.5-24.99 kg/m²** (N = 503)*	**BMI 25-29.99 kg/m²** (N = 827)*	**BMI≥30 kg/m²** (N = 476)*	**p-value#**
Age (years)	56.7 ± 5.5	57.9 ± 5.3	58.7 ± 5.4	<0.001
Sex (Male)	164 (33%)	460 (56%)	209 (44%)	<0.001
BMI	23.1 ± 1.5	27.2 ± 1.4	32.6 ± 3.3	<0.001
Smoking				
Current	30 (6.0%)	66 (8.0%)	33 (6.9%)	0.013
Never	294 (58%)	404 (49%)	238 (50%)	
Past	179 (36%)	357 (43%)	204 (43%)	
Height (cm)	167 ± 9	170 ± 9	167 ± 10	<0.001
Weight (kg)	64 ± 9	79 ± 9	95 ± 13	<0.001
Waist circumference (cm)	78 ± 7	91 ± 8	104 ± 9	<0.001
Systolic blood pressure (mmHg)	121 ± 14	128 ± 14	138 ± 16	<0.001
Diastolic blood pressure (mmHg)	72 ± 9	77 ± 9	81 ± 9	<0.001
HbA1c (%)	5.60 ± 0.25	5.60 ± 0.27	5.70 ± 0.29	<0.001
C-reactive protein (mg/L)	0.90 ± 3.09	1.50 ± 3.41	2.80 ± 5.86	<0.001
Alcohol consumption (std drinks/week)	6 ± 11	8 ± 14	7 ± 14	<0.001
Moderate physical activity (mins/week)	315 ± 732	210 ± 778	140 ± 684	<0.001

Vigorous physical activity (mins/week)	210 ± 552	210 ± 749	0 ± 616	<0.001
Blood pressure medication	37 (7.4%)	154 (19%)	138 (29%)	<0.001
Lipid-lowering medication	31 (6.2%)	127 (15%)	85 (18%)	<0.001
Cardiovascular disease	14 (2.8%)	57 (6.9%)	31 (6.5%)	0.004
* Median ± SD; n (%)				
# Kruskal-Wallis rank sum test; Pearson's Chi-squared test				

Quantification of Plasma Lipoproteins

A total of 112 lipoprotein parameters were quantified based on 1D NMR experiments as part of Bruker's IVDr experiment suite for blood plasma. This approach is termed Bruker's IVDr lipoprotein class and subclass Analysis (B.I.-LISA™) and lipid analytes include cholesterol, free cholesterol, phospholipids, triglycerides, apolipoproteins A1/A2/B100 and ratio B100/A1, in total plasma concentration and resolved for main lipoprotein classes and subclasses. Main classes of plasma-lipoproteins were defined as: high-density lipoprotein (HDL, density 1.063–1.210 kg/L), low-density lipoprotein (LDL, density 1.019–1.063 kg/L), intermediate-density lipoprotein (IDL, density 1.006–1.019 kg/L) and very low-density lipoprotein (VLDL, 0.950–1.006 kg/L). The main lipoprotein classes HDL, LDL, VLDL are further divided into different lipoprotein subclasses: (LDL-1: 1.019–1.031 kg/L, LDL-2: 1.031–1.034 kg/L, LDL-3: 1.034–1.037 kg/L, LDL-4: 1.037–1.040 kg/L, LDL-5: 1.040–1.044 kg/L, LDL-6: 1.044–1.063 kg/L), and the HDL subfractions into four density classes (HDL-1 1.063–1.100 kg/L, HDL-2 1.100–1.125 kg/L, HDL-3 1.112–1.125 kg/L, and HDL-4 1.125–1.210 kg/L), the VLDL sub-fractions divided into 5 density classes and density ranges are not supplied by Bruker.

Table S2. Annotation of the keys used by the Bruker IVDr Lipoprotein Subclass Analysis (B.I.-LISA ™) method. Abbreviations: LDL – low-density lipoprotein; HDL – high-density lipoprotein; VLDL – very low-density lipoprotein; IDL – intermediate-density lipoprotein.

Key	Class/subclass	Compound	Concentration unit
TPTG	Total Plasma	Triglycerides	mg/dL
TPCH	Total Plasma	Cholesterol	mg/dL
LDCH	LDL	Cholesterol	mg/dL
HDCH	HDL	Cholesterol	mg/dL
TPA1	Total Plasma	Apolipoprotein-A1	mg/dL
TPA2	Total Plasma	Apolipoprotein-A2	mg/dL
TPAB	Total Plasma	Apolipoprotein-B100	mg/dL
LDHD	Ratio LDL and HDL Cholesterol	LDL Cholesterol / HDL Cholesterol	-/-
ABA1	Ratio of Apolipoproteins B100 and A1	Apolipoprotein-B100 / Apolipoprotein-A1	-/-
TBPN	Apolipoprotein-B100 carrying particles	Particle Number	nmol/L
VLPN	VLDL	Particle Number	nmol/L
IDPN	IDL	Particle Number	nmol/L

Key	Class/subclass	Compound	Concentration unit
LDPN	LDL	Particle Number	nmol/L
L1PN	LDL-1	Particle Number	nmol/L
L2PN	LDL-2	Particle Number	nmol/L
L3PN	LDL-3	Particle Number	nmol/L
L4PN	LDL-4	Particle Number	nmol/L
L5PN	LDL-5	Particle Number	nmol/L
L6PN	LDL-6	Particle Number	nmol/L
VLTG	VLDL Class	Triglycerides	mg/dL
IDTG	IDL Class	Triglycerides	mg/dL
LDTG	LDL Class	Triglycerides	mg/dL
HDTG	HDL Class	Triglycerides	mg/dL
VLCH	VLDL Class	Cholesterol	mg/dL
IDCH	IDL Class	Cholesterol	mg/dL
LDCH	LDL Class	Cholesterol	mg/dL
HDCH	HDL Class	Cholesterol	mg/dL
VLFC	VLDL Class	Free Cholesterol	mg/dL
IDFC	IDL Class	Free Cholesterol	mg/dL
LDFC	LDL Class	Free Cholesterol	mg/dL
HDFC	HDL Class	Free Cholesterol	mg/dL
VLPL	VLDL Class	Phospholipids	mg/dL
IDPL	IDL Class	Phospholipids	mg/dL
LDPL	LDL Class	Phospholipids	mg/dL
HDPL	HDL Class	PhospholipidsI	mg/dL
HDA1	HDL Class	Apolipoprotein-A1	mg/dL
HDA2	HDL Class	Apolipoprotein-A2	mg/dL
VLAB	VLDL Class	Apolipoprotein-B100	mg/dL
IDAB	IDL Class	Apolipoprotein-B100	mg/dL
LDAB	LDL Class	Apolipoprotein-B100	mg/dL
V1TG	VLDL-1 Subclass	Triglycerides	mg/dL
V2TG	VLDL-2 Subclass	Triglycerides	mg/dL

Key	Class/subclass	Compound	Concentration unit
V3TG	VLDL-3 Subclass	Triglycerides	mg/dL
V4TG	VLDL-4 Subclass	Triglycerides	mg/dL
V5TG	VLDL-5 Subclass	Triglycerides	mg/dL
V1CH	VLDL-1 Subclass	Cholesterol	mg/dL
V2CH	VLDL-2 Subclass	Cholesterol	mg/dL
V3CH	VLDL-3 Subclass	Cholesterol	mg/dL
V4CH	VLDL-4 Subclass	Cholesterol	mg/dL
V5CH	VLDL-5 Subclass	Cholesterol	mg/dL
V1FC	VLDL-1 Subclass	Free Cholesterol	mg/dL
V2FC	VLDL-2 Subclass	Free Cholesterol	mg/dL
V3FC	VLDL-3 Subclass	Free Cholesterol	mg/dL
V4FC	VLDL-4 Subclass	Free Cholesterol	mg/dL
V5FC	VLDL-5 Subclass	Free Cholesterol	mg/dL
V1PL	VLDL-1 Subclass	Phospholipids	mg/dL
V2PL	VLDL-2 Subclass	Phospholipids	mg/dL
V3PL	VLDL-3 Subclass	Phospholipids	mg/dL
V4PL	VLDL-4 Subclass	Phospholipids	mg/dL
V5PL	VLDL-5 Subclass	Phospholipids	mg/dL
L1TG	LDL-1 Subclass	Triglycerides	mg/dL
L2TG	LDL-2 Subclass	Triglycerides	mg/dL
L3TG	LDL-3 Subclass	Triglycerides	mg/dL
L4TG	LDL-4 Subclass	Triglycerides	mg/dL
L5TG	LDL-5 Subclass	Triglycerides	mg/dL
L6TG	LDL-6 Subclass	Triglycerides	mg/dL
L1CH	LDL-1 Subclass	Cholesterol	mg/dL
L2CH	LDL-2 Subclass	Cholesterol	mg/dL
L3CH	LDL-3 Subclass	Cholesterol	mg/dL
L4CH	LDL-4 Subclass	Cholesterol	mg/dL
L5CH	LDL-5 Subclass	Cholesterol	mg/dL
L6CH	LDL-6 Subclass	Cholesterol	mg/dL

Key	Class/subclass	Compound	Concentration unit
L1FC	LDL-1 Subclass	Free Cholesterol	mg/dL
L2FC	LDL-2 Subclass	Free Cholesterol	mg/dL
L3FC	LDL-3 Subclass	Free Cholesterol	mg/dL
L4FC	LDL-4 Subclass	Free Cholesterol	mg/dL
L5FC	LDL-5 Subclass	Free Cholesterol	mg/dL
L6FC	LDL-6 Subclass	Free Cholesterol	mg/dL
L1PL	LDL-1 Subclass	Phospholipids	mg/dL
L2PL	LDL-2 Subclass	Phospholipids	mg/dL
L3PL	LDL-3 Subclass	Phospholipids	mg/dL
L4PL	LDL-4 Subclass	Phospholipids	mg/dL
L5PL	LDL-5 Subclass	Phospholipids	mg/dL
L6PL	LDL-6 Subclass	Phospholipids	mg/dL
L1AB	LDL-1 Subclass	Apolipoprotein-B100	mg/dL
L2AB	LDL-2 Subclass	Apolipoprotein-B100	mg/dL
L3AB	LDL-3 Subclass	Apolipoprotein-B100	mg/dL
L4AB	LDL-4 Subclass	Apolipoprotein-B100	mg/dL
L5AB	LDL-5 Subclass	Apolipoprotein-B100	mg/dL
L6AB	LDL-6 Subclass	Apolipoprotein-B100	mg/dL
H1TG	HDL-1 Subclass	Triglycerides	mg/dL
H2TG	HDL-2 Subclass	Triglycerides	mg/dL
H3TG	HDL-3 Subclass	Triglycerides	mg/dL
H4TG	HDL-4 Subclass	Triglycerides	mg/dL
H1CH	HDL-1 Subclass	Cholesterol	mg/dL
H2CH	HDL-2 Subclass	Cholesterol	mg/dL
H3CH	HDL-3 Subclass	Cholesterol	mg/dL
H4CH	HDL-4 Subclass	Cholesterol	mg/dL
H1FC	HDL-1 Subclass	Free Cholesterol	mg/dL
H2FC	HDL-2 Subclass	Free Cholesterol	mg/dL
H3FC	HDL-3 Subclass	Free Cholesterol	mg/dL
H4FC	HDL-4 Subclass	Free Cholesterol	mg/dL

Key	Class/subclass	Compound	Concentration unit
H1PL	HDL-1 Subclass	Phospholipids	mg/dL
H2PL	HDL-2 Subclass	Phospholipids	mg/dL
H3PL	HDL-3 Subclass	Phospholipids	mg/dL
H4PL	HDL-4 Subclass	Phospholipids	mg/dL
H1A1	HDL-1 Subclass	Apolipoprotein-A1	mg/dL
H2A1	HDL-2 Subclass	Apolipoprotein-A1	mg/dL
H3A1	HDL-3 Subclass	Apolipoprotein-A1	mg/dL
H4A1	HDL-4 Subclass	Apolipoprotein-A1	mg/dL
H1A2	HDL-1 Subclass	Apolipoprotein-A2	mg/dL
H2A2	HDL-2 Subclass	Apolipoprotein-A2	mg/dL
H3A2	HDL-3 Subclass	Apolipoprotein-A2	mg/dL
H4A2	HDL-4 Subclass	Apolipoprotein-A2	mg/dL

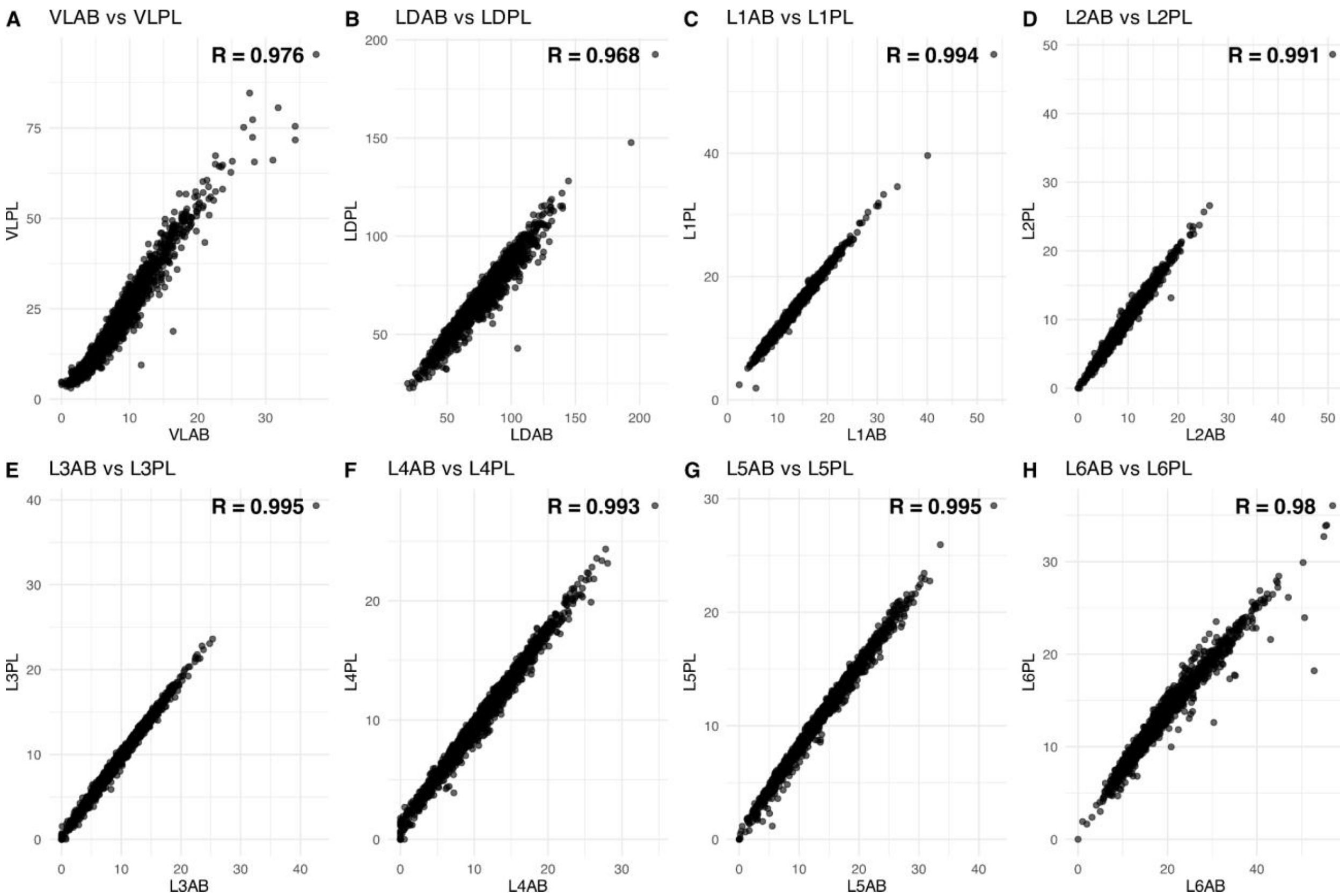

Figure S1. Linear correlation models of (A) VLAB versus VLPL, (B) LDAB versus LDPL, (C) L1AB vs L1PL, (D) L2AB versus L2PL, (E) L3AB versus L3PL, (F) L4AB versus L4PL, (G) L5AB versus L5PL (H) L6AB versus L6PL.

Table S3. Summary of NMR IVDr derived lipoproteins stratified by BMI categories.

	**Healthy weight**, N = 503[1]	**Overweight**, N = 827[1]	**Obese**, N = 476[1]	**p-value**[2]
TPTG (mg/dL)	72 ± 48	100 ± 77	117 ± 64	<0.001
TPCH (mg/dL)	210 ± 36	214 ± 42	211 ± 41	0.3
LDCH (mg/dL)	118 ± 28	122 ± 34	121 ± 34	0.3
HDCH (mg/dL)	66 ± 15	57 ± 14	54 ± 12	<0.001
TPA1 (mg/dL)	162 ± 26	149 ± 24	145 ± 24	<0.001
TPA2 (mg/dL)	34.7 ± 5.8	34.8 ± 5.4	33.9 ± 5.5	0.063
TPAB (mg/dL)	84 ± 18	91 ± 23	93 ± 22	<0.001
LDHD (-/-)	1.79 ± 0.57	2.12 ± 0.71	2.24 ± 0.71	<0.001
ABA1 (-/-)	0.52 ± 0.15	0.61 ± 0.18	0.65 ± 0.18	<0.001
TBPN (nmol/L)	1,518 ± 335	1,660 ± 417	1,689 ± 404	<0.001
VLPN (nmol/L)	93 ± 65	138 ± 81	153 ± 75	<0.001
IDPN (nmol/L)	68 ± 37	87 ± 43	97 ± 40	<0.001
LDPN (nmol/L)	1,327 ± 294	1,392 ± 371	1,394 ± 371	<0.001
L1PN (nmol/L)	242 ± 65	233 ± 77	227 ± 68	<0.001

[OFFICIAL

	Healthy weight, N = 503[1]	Overweight, N = 827[1]	Obese, N = 476[1]	p-value[2]
L2PN (nmol/L)	196 ± 63	168 ± 73	165 ± 63	<0.001
L3PN (nmol/L)	199 ± 63	183 ± 78	179 ± 76	<0.001
L4PN (nmol/L)	183 ± 89	210 ± 92	215 ± 93	<0.001
L5PN (nmol/L)	179 ± 88	235 ± 106	245 ± 104	<0.001
L6PN (nmol/L)	291 ± 102	323 ± 140	328 ± 134	<0.001
VLTG (mg/dL)	39 ± 36	61 ± 56	74 ± 48	<0.001
IDTG (mg/dL)	4 ± 8	8 ± 14	11 ± 11	<0.001
LDTG (mg/dL)	17.0 ± 4.7	18.1 ± 6.8	18.7 ± 5.9	<0.001
HDTG (mg/dL)	10.4 ± 3.7	10.7 ± 4.1	10.9 ± 3.4	0.001
VLCH (mg/dL)	11 ± 11	17 ± 15	19 ± 14	<0.001
IDCH (mg/dL)	8 ± 6	12 ± 7	14 ± 7	<0.001
VLFC (mg/dL)	5.7 ± 4.3	8.5 ± 5.5	9.4 ± 5.1	<0.001
IDFC (mg/dL)	2.40 ± 1.70	3.39 ± 2.05	3.76 ± 1.91	<0.001
LDFC (mg/dL)	34 ± 7	34 ± 9	33 ± 9	0.12
HDFC (mg/dL)	14.8 ± 4.3	11.7 ± 4.0	11.0 ± 3.6	<0.001

[OFFICIAL

	Healthy weight, N = 503[1]	Overweight, N = 827[1]	Obese, N = 476[1]	p-value[2]
VLPL (mg/dL)	13 ± 9	19 ± 12	22 ± 11	<0.001
IDPL (mg/dL)	4.9 ± 3.2	6.5 ± 4.4	7.2 ± 3.9	<0.001
LDPL (mg/dL)	67 ± 14	69 ± 17	68 ± 16	0.4
HDPL (mg/dL)	89 ± 20	78 ± 18	75 ± 17	<0.001
HDA1 (mg/dL)	164 ± 27	150 ± 25	146 ± 25	<0.001
HDA2 (mg/dL)	34.9 ± 5.5	35.2 ± 5.3	34.4 ± 5.3	0.2
VLAB (mg/dL)	5.1 ± 3.6	7.6 ± 4.4	8.4 ± 4.1	<0.001
IDAB (mg/dL)	3.75 ± 2.04	4.81 ± 2.34	5.34 ± 2.22	<0.001
LDAB (mg/dL)	73 ± 16	77 ± 20	77 ± 20	<0.001
V1TG (mg/dL)	15 ± 19	27 ± 31	35 ± 25	<0.001
V2TG (mg/dL)	6 ± 8	10 ± 12	11 ± 11	<0.001
V3TG (mg/dL)	6 ± 7	9 ± 9	10 ± 8	<0.001
V4TG (mg/dL)	6.2 ± 4.2	8.6 ± 4.9	9.6 ± 4.8	<0.001
V5TG (mg/dL)	2.63 ± 0.86	2.93 ± 0.88	3.19 ± 0.91	<0.001
V1CH (mg/dL)	2.6 ± 3.9	4.6 ± 5.8	5.8 ± 5.0	<0.001

[OFFICIAL

	**Healthy weight**, N = 503[1]	**Overweight**, N = 827[1]	**Obese**, N = 476[1]	**p-value**[2]
V2CH (mg/dL)	1.34 ± 2.09	2.17 ± 2.90	2.43 ± 2.70	<0.001
V3CH (mg/dL)	1.57 ± 2.33	2.77 ± 3.07	3.15 ± 2.81	<0.001
V4CH (mg/dL)	3.3 ± 2.8	4.8 ± 3.2	5.3 ± 3.1	<0.001
V5CH (mg/dL)	1.49 ± 0.71	1.71 ± 0.70	1.81 ± 0.68	<0.001
V1FC (mg/dL)	0.89 ± 1.50	1.83 ± 2.24	2.38 ± 1.98	<0.001
V2FC (mg/dL)	0.47 ± 0.94	0.87 ± 1.27	1.02 ± 1.18	<0.001
V3FC (mg/dL)	0.56 ± 0.98	1.04 ± 1.37	1.27 ± 1.24	<0.001
V4FC (mg/dL)	1.15 ± 1.25	1.80 ± 1.37	2.03 ± 1.33	<0.001
V5FC (mg/dL)	0.54 ± 0.44	0.69 ± 0.45	0.77 ± 0.44	<0.001
V1PL (mg/dL)	2.6 ± 3.4	4.7 ± 5.3	6.1 ± 4.4	<0.001
V2PL (mg/dL)	1.73 ± 2.00	2.74 ± 2.87	3.22 ± 2.57	<0.001
V3PL (mg/dL)	2.05 ± 2.09	3.14 ± 2.76	3.64 ± 2.53	<0.001
V4PL (mg/dL)	3.29 ± 2.18	4.61 ± 2.47	5.05 ± 2.39	<0.001
V5PL (mg/dL)	1.64 ± 0.79	1.96 ± 0.78	2.13 ± 0.76	<0.001
L1TG (mg/dL)	5.38 ± 2.03	5.56 ± 2.76	5.82 ± 2.15	<0.001

[OFFICIAL

	Healthy weight, N = 503[1]	Overweight, N = 827[1]	Obese, N = 476[1]	p-value[2]
L2TG (mg/dL)	2.42 ± 0.73	2.24 ± 0.93	2.24 ± 0.78	<0.001
L3TG (mg/dL)	2.67 ± 0.62	2.67 ± 0.77	2.75 ± 0.74	0.2
L4TG (mg/dL)	2.06 ± 1.02	2.35 ± 1.19	2.54 ± 1.17	<0.001
L5TG (mg/dL)	1.83 ± 0.98	2.25 ± 1.40	2.46 ± 1.37	<0.001
L6TG (mg/dL)	3.50 ± 1.42	3.84 ± 2.02	4.08 ± 1.78	<0.001
L1CH (mg/dL)	25 ± 7	25 ± 8	24 ± 8	<0.001
L2CH (mg/dL)	20 ± 7	16 ± 8	16 ± 7	<0.001
L3CH (mg/dL)	19 ± 7	18 ± 8	17 ± 8	<0.001
L4CH (mg/dL)	17 ± 8	19 ± 8	20 ± 8	<0.001
L5CH (mg/dL)	15 ± 7	19 ± 9	20 ± 8	<0.001
L6CH (mg/dL)	20 ± 7	22 ± 9	22 ± 9	<0.001
L1FC (mg/dL)	7.48 ± 2.04	7.07 ± 2.45	6.79 ± 2.22	<0.001
L2FC (mg/dL)	6.22 ± 2.11	5.08 ± 2.41	4.81 ± 2.07	<0.001
L3FC (mg/dL)	6.20 ± 1.85	5.41 ± 2.30	5.03 ± 2.16	<0.001
L4FC (mg/dL)	5.22 ± 1.83	5.38 ± 2.05	5.53 ± 2.06	0.084

[OFFICIAL

	Healthy weight, N = 503[1]	Overweight, N = 827[1]	Obese, N = 476[1]	p-value[2]
L5FC (mg/dL)	4.38 ± 1.67	5.11 ± 1.98	5.11 ± 1.94	<0.001
L6FC (mg/dL)	5.17 ± 1.69	5.52 ± 2.06	5.37 ± 1.94	0.005
L1PL (mg/dL)	14.6 ± 3.7	14.1 ± 4.3	13.8 ± 3.9	<0.001
L2PL (mg/dL)	11.2 ± 3.5	9.3 ± 4.0	9.0 ± 3.5	<0.001
L3PL (mg/dL)	10.8 ± 3.2	9.9 ± 4.0	9.6 ± 3.9	<0.001
L4PL (mg/dL)	9.6 ± 4.0	10.6 ± 4.2	10.9 ± 4.3	<0.001
L5PL (mg/dL)	8.1 ± 3.6	10.4 ± 4.3	10.8 ± 4.2	<0.001
L6PL (mg/dL)	11.6 ± 3.5	12.5 ± 4.5	12.4 ± 4.4	<0.001
L1AB (mg/dL)	13.3 ± 3.6	12.8 ± 4.2	12.5 ± 3.8	<0.001
L2AB (mg/dL)	10.8 ± 3.4	9.2 ± 4.0	9.1 ± 3.5	<0.001
L3AB (mg/dL)	10.9 ± 3.4	10.1 ± 4.3	9.9 ± 4.2	<0.001
L4AB (mg/dL)	10.1 ± 4.9	11.6 ± 5.0	11.9 ± 5.1	<0.001
L5AB (mg/dL)	9.9 ± 4.9	12.9 ± 5.9	13.5 ± 5.7	<0.001
L6AB (mg/dL)	16 ± 6	18 ± 8	18 ± 7	<0.001
H1TG (mg/dL)	3.50 ± 1.91	2.79 ± 2.06	2.80 ± 1.50	<0.001

[OFFICIAL

	Healthy weight, N = 503[1]	Overweight, N = 827[1]	Obese, N = 476[1]	p-value[2]
H2TG (mg/dL)	1.58 ± 0.73	1.57 ± 0.83	1.65 ± 0.71	0.025
H3TG (mg/dL)	1.87 ± 0.80	2.13 ± 0.88	2.26 ± 0.79	<0.001
H4TG (mg/dL)	3.38 ± 1.25	4.15 ± 1.24	4.27 ± 1.06	<0.001
H1CH (mg/dL)	20 ± 11	13 ± 9	12 ± 7	<0.001
H2CH (mg/dL)	9.72 ± 3.15	7.61 ± 2.93	7.40 ± 2.60	<0.001
H3CH (mg/dL)	11.60 ± 2.50	10.59 ± 2.62	10.38 ± 2.68	<0.001
H4CH (mg/dL)	22.8 ± 3.9	23.7 ± 4.1	23.0 ± 3.8	<0.001
H1FC (mg/dL)	5.12 ± 2.61	3.15 ± 2.14	2.78 ± 1.75	<0.001
H2FC (mg/dL)	2.15 ± 0.81	1.68 ± 0.78	1.57 ± 0.74	<0.001
H3FC (mg/dL)	2.49 ± 0.71	2.30 ± 0.73	2.20 ± 0.76	<0.001
H4FC (mg/dL)	4.31 ± 1.01	4.48 ± 1.06	4.29 ± 1.02	0.002
H1PL (mg/dL)	24 ± 13	15 ± 11	14 ± 8	<0.001
H2PL (mg/dL)	14.6 ± 4.6	12.1 ± 4.4	11.7 ± 4.2	<0.001
H3PL (mg/dL)	17.8 ± 4.2	16.9 ± 4.2	16.8 ± 4.5	<0.001
H4PL (mg/dL)	30.6 ± 5.2	32.0 ± 5.0	31.7 ± 4.6	<0.001

[OFFICIAL

	**Healthy weight**, N = 503[1]	**Overweight**, N = 827[1]	**Obese**, N = 476[1]	**p-value**[2]
H1A1 (mg/dL)	29 ± 19	17 ± 15	14 ± 12	<0.001
H2A1 (mg/dL)	21.2 ± 5.4	18.6 ± 5.2	18.2 ± 4.9	<0.001
H3A1 (mg/dL)	29 ± 6	28 ± 6	27 ± 7	<0.001
H4A1 (mg/dL)	82 ± 12	87 ± 12	86 ± 11	<0.001
H1A2 (mg/dL)	2.96 ± 1.79	1.96 ± 1.51	1.79 ± 1.24	<0.001
H2A2 (mg/dL)	3.67 ± 1.22	3.28 ± 1.19	3.24 ± 1.19	<0.001
H3A2 (mg/dL)	6.65 ± 1.72	6.66 ± 1.67	6.66 ± 1.76	>0.9
H4A2 (mg/dL)	21.1 ± 4.3	23.0 ± 4.0	22.4 ± 3.5	<0.001

[1] n (%); Median ± SD

[2] Fisher's exact test; Kruskal-Wallis rank sum test

[OFFICIAL

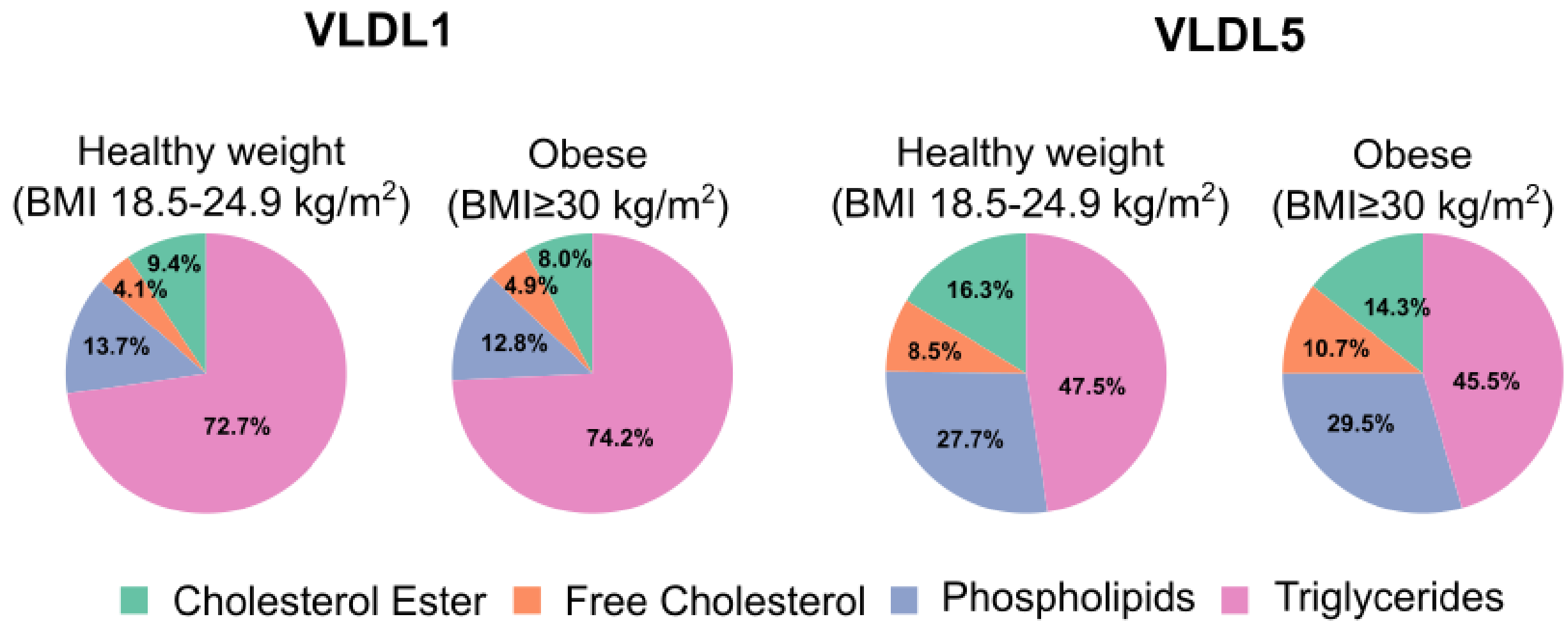

Figure S2. VLDL subclasses 1 and 5 compositions by BMI of 18.5-24.9 kg/m^2 and BMI≥30kg/m^2. CE = Cholesterol Ester, FC = Free Cholesterol, PL = Phospholipid, TG = Triglycerides.

Table S4. Adjusted *p*-values for comparisons of cholesterol ester, free cholesterol, phospholipid and triglyceride proportions in those individuals in the healthy weight group vs the obese group in subfractions VLDL1 and VLDL5.

Particle	Subfraction	Adjusted p-values
VLDL1	CE	0.032
VLDL1	FC	8.43×10^{-9}
VLDL1	PL	0.001
VLDL1	TG	ns
VLDL5	CE	6.63×10^{-17}
VLDL5	FC	7.61×10^{-13}
VLDL5	PL	1.49×10^{-16}
VLDL5	TG	8.06×10^{-5}

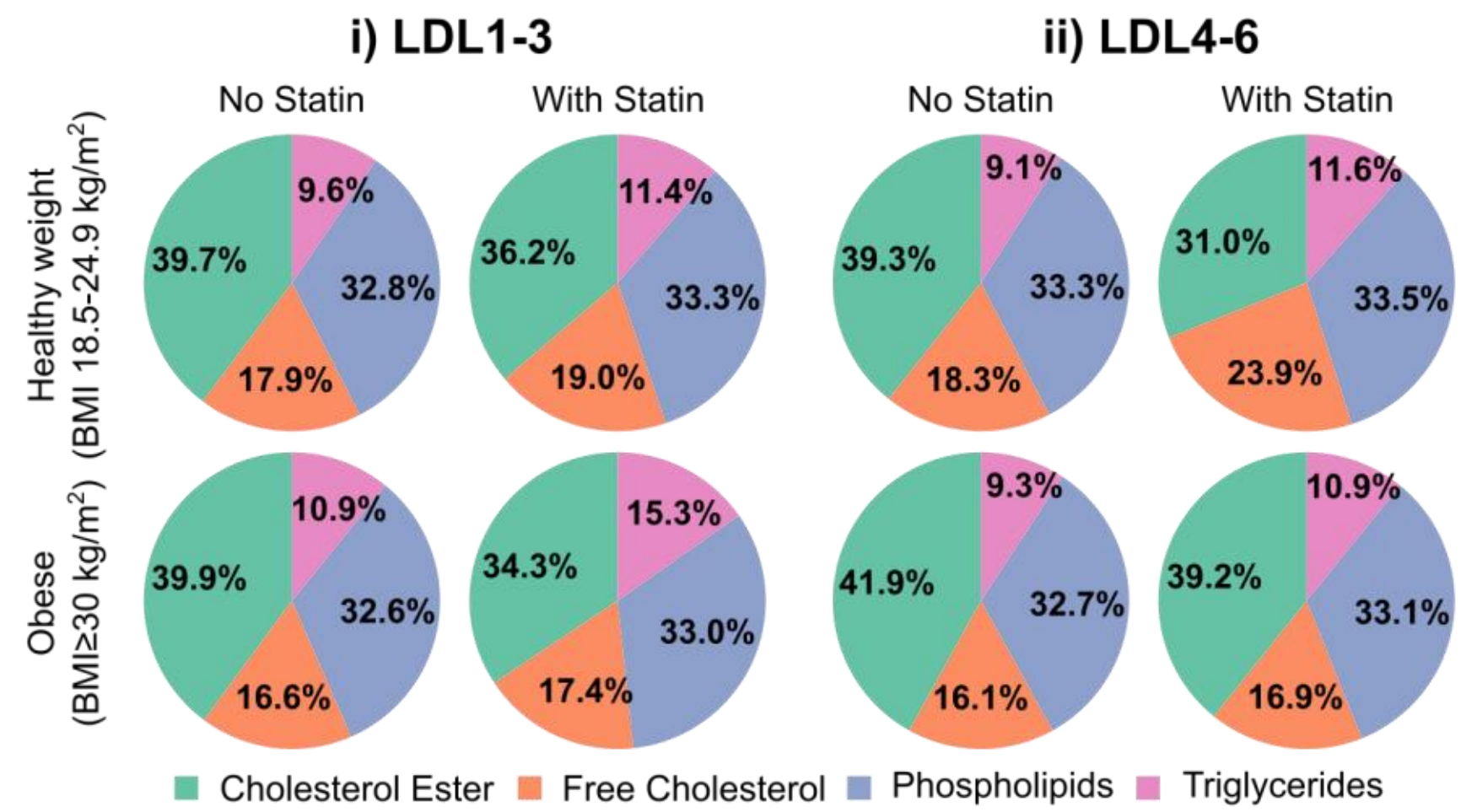

Figure S3. The proportion of the total in LDL1 to LDL3 (L1+2+3) with respect to LDL4 to LDL6 (L4+5+6) in BMI 18.5-24.9kg/m^2 vs. BMI ≥ 30 kg/m^2 (adjusted for particle number) further stratified to statin vs. no statin use t. CE = Cholesterol Ester, FC = Free Cholesterol, PL = Phospholipid, TG = Triglycerides.

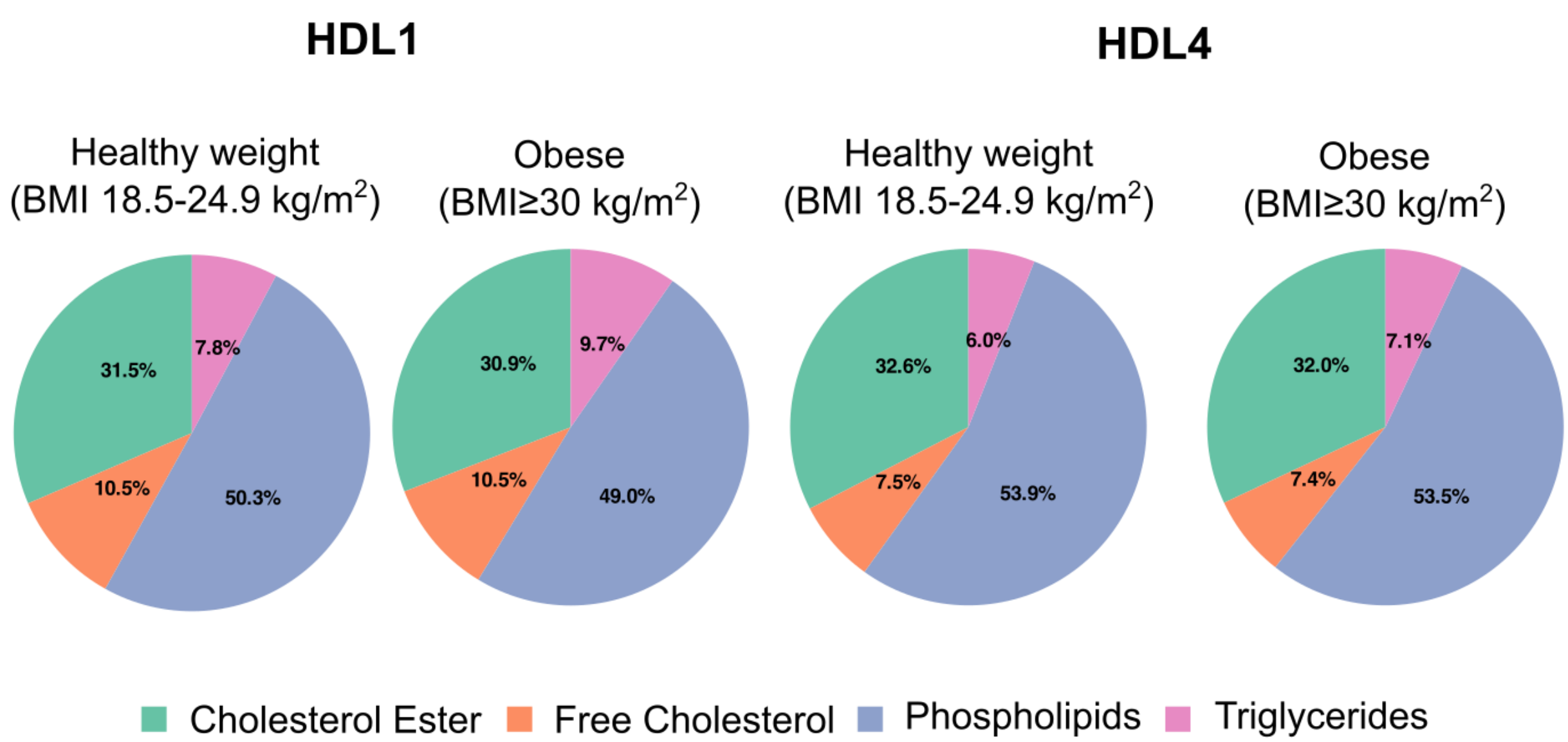

Figure S4. HDL composition of subclasses 1 and 4 by BMI of 18.5-24.9<25 kg/m^2 and BMI≥>30Kkg/m^2. CE = Cholesterol Ester, FC = Free Cholesterol, PL = Phospholipid, TG = Triglycerides.

Table S5. Adjusted *p*-values for comparisons of cholesterol ester, free cholesterol, phospholipid and triglyceride proportions in in the healthy weight group vs the obese group in subfractions HDL1 and HDL4.

Particle	Subfraction	Adjusted p-values
HDL1	CE	ns
HDL1	FC	1.83×10^{-14}
HDL1	PL	4.00×10^{-14}
HDL1	TG	4.64×10^{-24}
HDL4	CE	1.02×10^{-6}
HDL4	FC	4.38×10^{8}
HDL4	PL	6.14×10^{-4}
HDL4	TG	7.44×10^{-30}

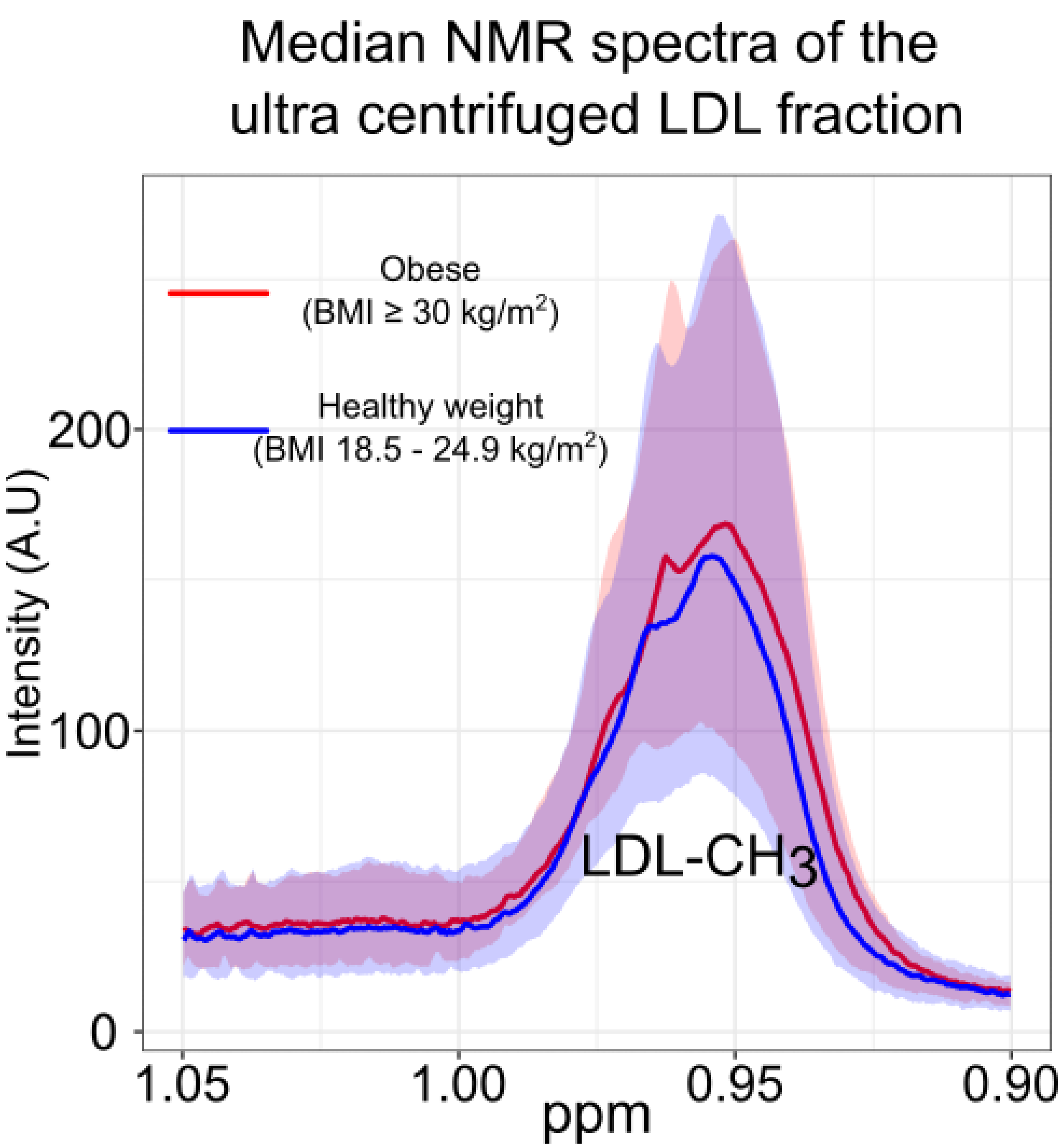

Figure S5. Zoomed NMR spectral region of a 1D ^{1}H experiment showing the $-CH_3$ of the LDL lipoprotein for healthy weight and obese weight individuals.

Healthy weight (BMI 18.5 - 24.9 kg/m²)

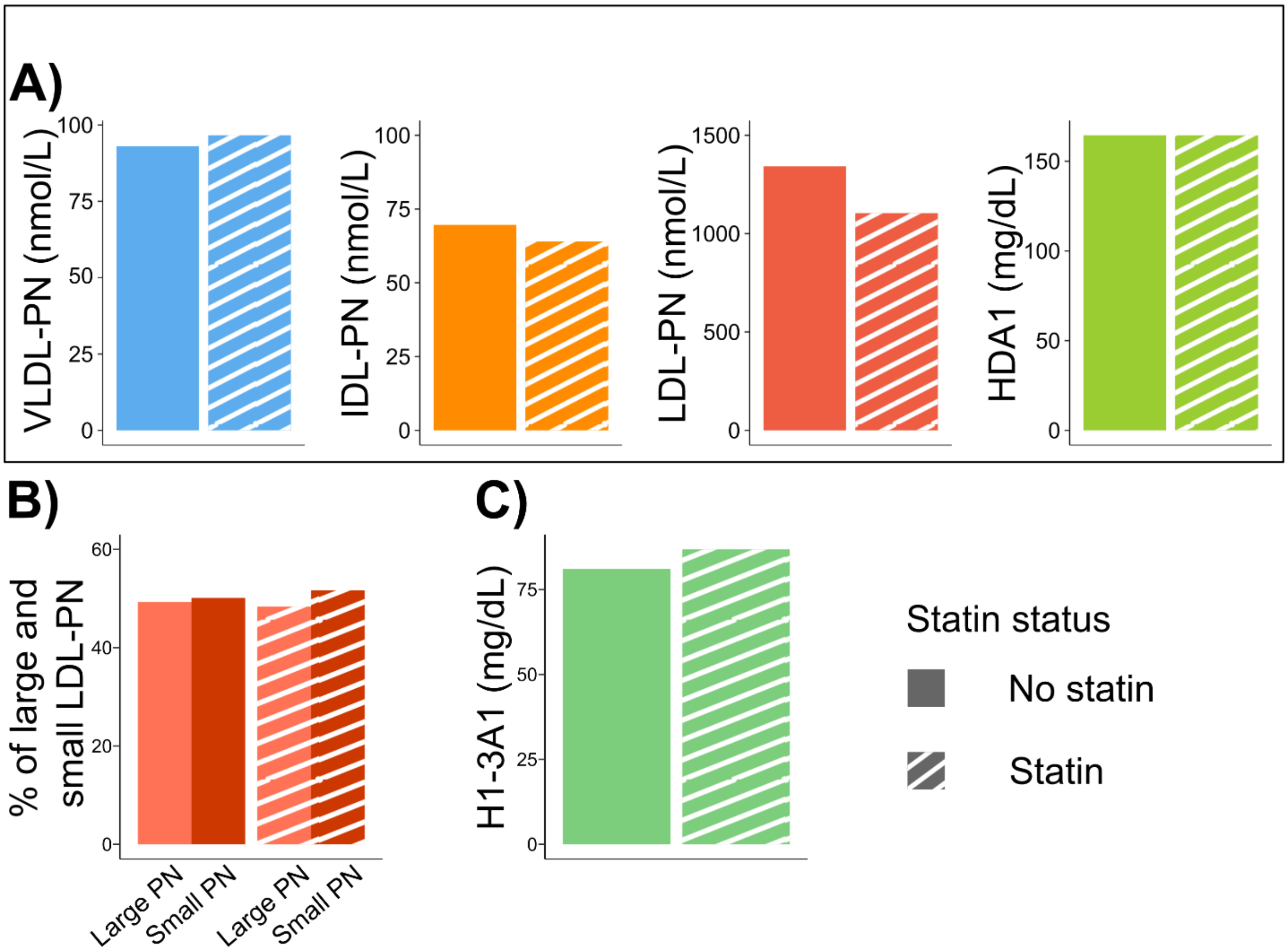

H or HD = HDL, A1 = Apolipoprotein-A1, PN = Particle Number

Figure S6. Bar plots showing the effect of statin intake in particle number distribution for the healthy weight individuals whose BMI in 18.5 - 24.9 kg/m² ranges. A) VLDL, IDL, LDL and HDL. (B) Large vs. small LDL . (C) HDL1-HDL3.

[OFFICIAL

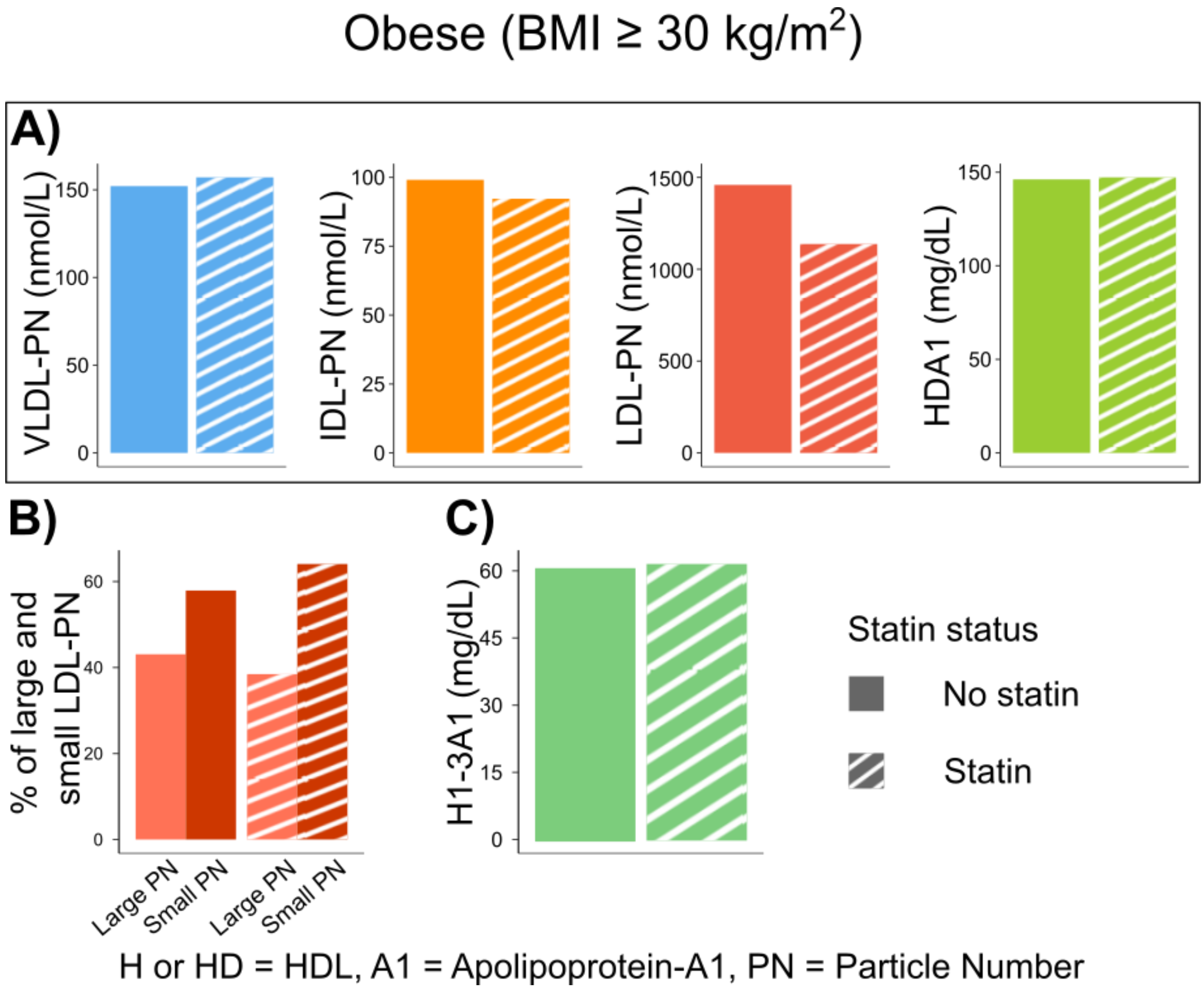

Figure S7. Bar plots showing the effect of statin intake in particle number distribution for the obese individuals whose BMI ≥ 30 kg/m^2. A) VLDL, IDL, LDL and HDL. B) Large vs. small LDL. C) HDL1-HDL3.

Table S6. The adjusted *p*-values for the comparison of the effect of statin intake on particle number of Total VLDL (A), IDL (B) and HDL (C) (particle number had been substituted by the amount of phospholipid for VLDL and for Apo-A1 for HDL) for individuals with BMI 18.5-24.9 kg/m^2

Variable	Statin Intake Effect on BMI 18.5-24.9 kg/m^2		p-value
	No Statin [median(IQR)]	Statin [median(IQR)]	
H1A1	29.365 (17.20–43.80)	30.64 (19.45–43.39)	ns
H2A1	21.155 (18.02–25.08)	22.67 (18.68–24.81)	ns

[OFFICIAL

H3A1	29.23 (25.79–33.38)	29.51 (27.50–33.24)	ns
H4A1	82.5 (73.87–90.45)	79.75 (71.64–87.06)	ns
H1-3A1	81.07 (26.83)	86.85 (28.72)	ns
HDA1	164.44 (145.82–183.57)	164.42 (147.17–185.12)	ns
IDPN	69.63 (47.90–90.79)	64.00 (44.43–92.84)	ns
LDPN	1342.275 (1149.24–1530.86)	1103.91 (987.07–1233.16)	1.89×10^{-5}
L1PN	243.42 (209.85–283.13)	205.34 (190.99–244.54)	0.005
L2PN	197.35 (160.44–239.32)	149.07 (118.98–184.34)	7×10^{-5}
L3PN	202.035 (162.00–239.35)	134.42 (117.92–160.70)	1.37×10^{-7}
L4PN	185.85 (127.62–244.49)	120.51 (66.12–155.75)	6.82×10^{-5}
L5PN	182.09 (131.37–244.50)	143.53 (107.46–202.55)	ns
L6PN	289.5 (242.26–349.19)	306.9 (238.19–352.61)	ns
L1-3PN	634.82 (168.67)	478.65 (150.26)	9.44×10^{-6}
L4-6PN	656.55 (223.71)	597.29 (182.31)	0.032
V1PL	2.615 (1.53–4.08)	2.2 (1.44–3.92)	ns
V2PL	1.74 (1.19–2.72)	1.70 (1.10–2.52)	ns
V3PL	2.05 (1.24–3.10)	1.97 (0.99–3.00)	ns
V4PL	3.30 (2.33–4.62)	3.19 (2.42–4.68)	ns
V5PL	1.64 (1.23–2.13)	1.64 (1.26–2.46)	ns
VLPL	12.88 (9.08–18.23)	12.69 (9.20–17.91)	ns

[OFFICIAL

Table S7. The adjusted *p*-values for the comparison of the effect of statin intake on particle number of Total VLDL (A), IDL (B) and HDL (C) (particle number had been substituted by the amount of phospholipid for VLDL and for Apo-A1 for HDL) for individuals with BMI≧30 kg/m²

Variable	Statin Intake Effect on BMI≥30 kg/m2		p-value
	No Statin [median(IQR)]	Statin [median(IQR)]	
H1A1	14.20 (9.48–21.94)	15.38 (11.31–20.89)	ns
H2A1	18.08 (15.15–21.14)	18.49 (15.15–21.70)	ns
H3A1	27.35 (22.90–32.03)	27.89 (22.87–32.78)	ns
H4A1	85.30 (77.94–92.71)	86.89 (77.31–95.41)	ns
H1-3A1	60.31 (21.09)	61.30 (19.27)	ns
HDA1	146.13 (129.63–161.17)	147.22 (132.87–170.00)	ns
IDPN	99.10 (72.72–124.34)	92.24 (63.06–113.16)	ns
LDPN	1458.88 (1241.24–1685.47)	1137.09 (875.14–1299.92)	2.53×10^{-16}
L1PN	236.16 (194.84–279.70)	174.06 (137.50–232.13)	9.64×10^{-13}
L2PN	177.44 (135.21–219.19)	116.83 (83.81–152.74)	2.19×10^{-15}
L3PN	190.43 (145.13–237.88)	117.34 (74.75–158.48)	1.13×10^{-14}
L4PN	230.11 (177.38–284.75)	161.98 (99.10–211.40)	7.69×10^{-10}
L5PN	254.34 (195.18–336.18)	206.85 (152.09–284.23)	5.7×10^{-5}
L6PN	336.96 (262.36–439.53)	300.60 (237.30–392.65)	0.009
L1-3PN	605.34 (175.85)	396.30 (160.50)	5.84×10^{-17}
L4-6PN	825.24 (277.16)	679.19 (223.56)	9.09×10^{-7}
V1PL	6.09 (3.83–8.93)	6.03 (3.91–9.31)	ns
V2PL	3.24 (1.98–4.90)	3.10 (2.20–5.08)	ns
V3PL	3.6 (2.18–5.33)	3.72 (2.68–5.78)	ns

[OFFICIAL

V4PL	5.02 (3.53–6.66)	5.31 (3.91–7.10)	ns
V5PL	2.11 (1.63–2.57)	2.15 (1.72–2.73)	ns
VLPL	21.70 (15.66–29.30)	22.48 (15.79–30.94)	ns

Table S8. Comparison of the composition of the LDL subfractions in the healthy weight individuals taking and not taking statin medication.

Variable	Composition	Average total of individual composition(%)		p-value
		No Statin - BMI 18.5-24.9 kg/m^2 (SD)	Statin - BMI 18.5-24.9 kg/m^2 (SD)	
LDL1-LDL3	CE	39.7 (3.7)	36.2 (3.3)	2.25×10^{-6}
LDL1-LDL3	FC	17.9 (1.9)	19.0 (2.3)	NS
LDL1-LDL3	PL	32.8 (0.9)	33.3 (0.6)	0.010
LDL1-LDL3	TG	9.6 (4.0)	11.4 (3.0)	0.0003
LDL4-LDL6	CE	39.3 (16.0)	31.0 (28.4)	0.004
LDL4-LDL6	FC	18.3 (13.3)	23.9 (23.5)	NS
LDL4-LDL6	PL	33.3 (2.1)	33.5 (2.5)	NS
LDL4-LDL6	TG	9.10 (5.1)	11.6 (7.0)	0.008

Table S9. Comparison of the composition of the LDL subfractions in the obese weight individuals taking and not taking statin medication.

Variable	Composition	Average total of individual composition(%)		p-value
		No Statin - BMI $\geq$30 kg/m^2 (SD)	Statin - BM$\geq$30 kg/m^2 (SD)	
LDL1-LDL3	CE	39.9 (4.4)	34.3 (8.0)	2.80×10^{-46}
LDL1-LDL3	FC	16.6 (2.1)	17.4 (3.6)	1.38×10^{-7}
LDL1-LDL3	PL	32.6 (1.3)	33.0 (3.7)	2.14×10^{-32}
LDL1-LDL3	TG	10.9 (5.1)	15.3 (11.9)	1.19×10^{-21}

[OFFICIAL

LDL4-LDL6	CE	41.9 (6.0)	39.2 (3.7)	2.94 x 10^{-17}
LDL4-LDL6	FC	16.1 (2.8)	16.9 (2.7)	NS
LDL4-LDL6	PL	32.7 (1.5)	33.1 (2.3)	1.18 x 10^{-8}
LDL4-LDL6	TG	9.3 (3.9)	10.9 (6.4)	3.08 x 10^{-11}

Table S10. BMI differences in particle number distribution for VLDL, IDL, LDL and HDL and their corresponding adjusted p-values.

	**Median (IQR)**		
**Particle Number**	**BMI 18.5-24.9 kg/m²<br>[median(IQR)]**	**BMI ≥30 kg/m²<br>[median(IQR)]**	***p*-value**
IDPN	68.11 (47.70–90.84)	96.98 (71.91–123.46)	4.12 x 10^{-27}
LDPN	1326.88 (1133.76–1519.65)	1394.02 (1149.62–1638.83)	0.05
L1PN	242.29 (205.89–281.76)	226.72 (182.69–271.68)	0.0042
L2PN	195.54 (157.96–238.09)	165.33 (119.81–213.30)	7.4 x 10^{-13}
L3PN	198.83 (156.82–235.44)	179.1 (124.56–228.46)	0.0004
L4PN	182.87 (121.88–241.00)	215.48 (159.35–275.60)	1.16 x 10^{-7}
L5PN	179.43 (130.42–244.40)	245.30 (187.25–326.73)	2 x 10^{-21}
L6PN	290.62 (242.12–349.43)	327.77 (257.48–429.07)	2.16 x 10^{-6}
L1-3PN	626.05 (537.78–746.13)	565.52 (437.96–706.60)	2.38 x 10^{-7}
L4-6PN	648.52 (524.98–803.99)	801.87 (637.25–1021.58)	2.52 x 10^{-16}
L1-3:4-6PN	0.98 (0.76–1.24)	0.71 (0.51–0.96)	4.46 x 10^{-27}
L2:5PN	1.09 (0.74–1.63)	0.66 (0.43–0.99)	6.79 x 10^{-31}
VLPL	12.85 (9.09–18.24)	21.77 (15.67–29.84)	2.59 x 10^{-43}
V1PL	2.57 (1.52–4.08)	6.08 (3.87–8.96)	6.6 x 10^{-58}
V2PL	1.73 (1.19–2.71)	3.22 (2.04–4.93)	1.42 x 10^{-34}
V3PL	2.05 (1.21–3.10)	3.63 (2.27–5.41)	1.73 x 10^{-30}
V4PL	3.29 (2.35–4.62)	5.04 (3.66–6.71)	1.02 x 10^{-30}
V5PL	1.64 (1.23–2.14)	2.12 (1.65–2.58)	6.87 x 10^{-20}
HDA1	164.42 (145.87–183.73)	146.27 (130.15–162.87)	4.51 x 10^{-21}
H1A1	29.48 (17.31–43.81)	14.37 (9.67–21.60)	5.34 x 10^{-42}
H2A1	21.16 (18.05–25.08)	18.15 (15.14–21.32)	5.81 x 10^{-19}

[OFFICIAL

	Median (IQR)		
**Particle Number**	**BMI 18.5-24.9 kg/m^2 [median(IQR)]**	**BMI $\geq$30 kg/m^2 [median(IQR)]**	***p*-value**
IDPN	68.11 (47.70–90.84)	96.98 (71.91–123.46)	4.12 x 10^{-27}
LDPN	1326.88 (1133.76–1519.65)	1394.02 (1149.62–1638.83)	0.05
L1PN	242.29 (205.89–281.76)	226.72 (182.69–271.68)	0.0042
L2PN	195.54 (157.96–238.09)	165.33 (119.81–213.30)	7.4 x 10^{-13}
L3PN	198.83 (156.82–235.44)	179.1 (124.56–228.46)	0.0004
L4PN	182.87 (121.88–241.00)	215.48 (159.35–275.60)	1.16 x 10^{-7}
L5PN	179.43 (130.42–244.40)	245.30 (187.25–326.73)	2 x 10^{-21}
H3A1	29.36 (25.83–33.39)	27.39 (22.89–32.15)	0.0004
H4A1	82.37 (73.80–90.27)	85.63 (77.81–93.51)	0.0011
H1-3A1	81.17 (63.45–101.88)	60.71 (48.98–74.45)	3.5 x 10^{-32}
H1-3:4A1	0.96 (0.73–1.29)	0.69 (0.58–0.89)	7.27 x 10^{-34}
H1:4A1	0.34 (0.20–0.56)	0.17 (0.11–0.27)	1.45 x 10^{-40}

[OFFICIAL

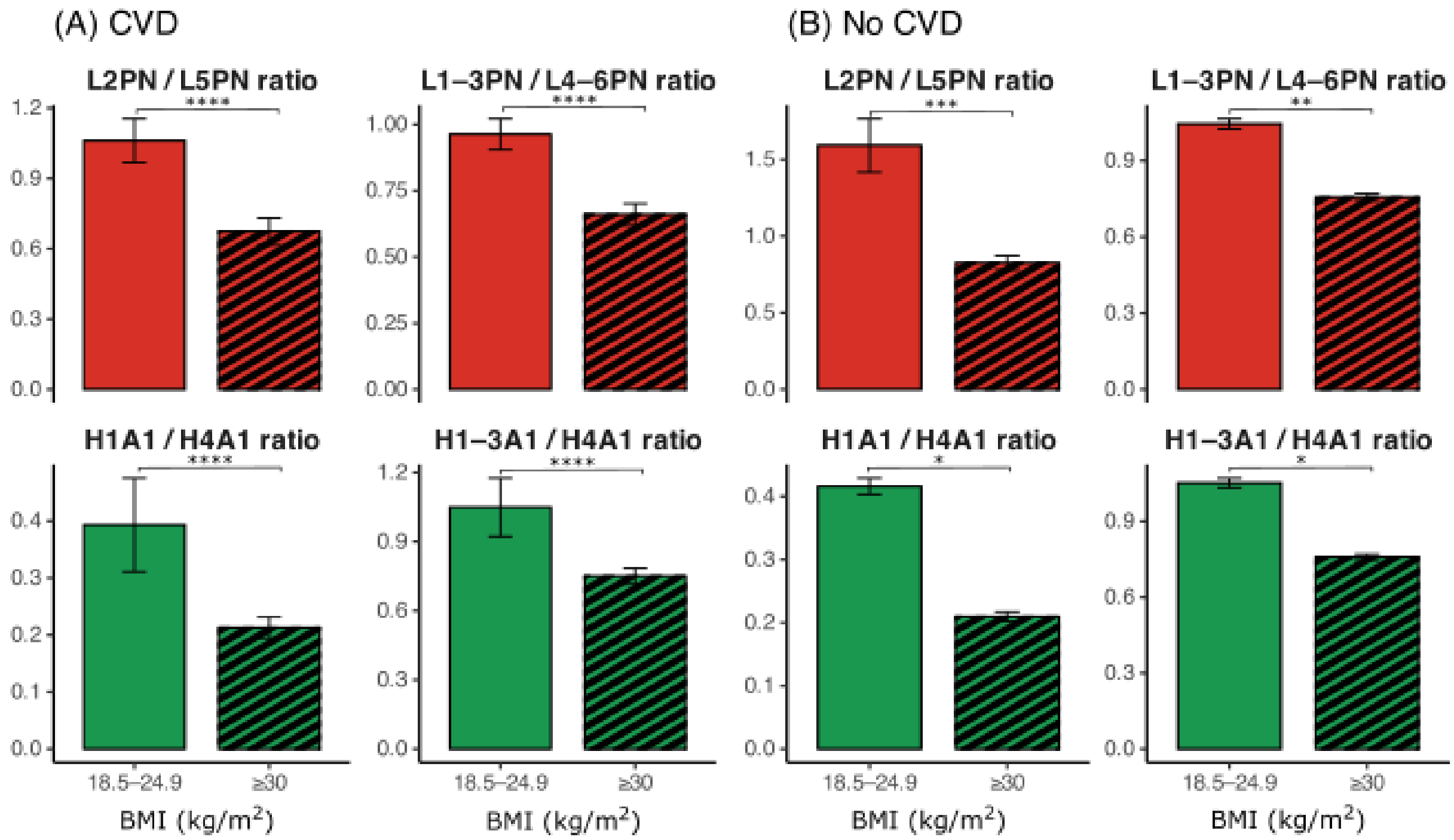

Figure S8. Bar plots showing BMI induced differences in the ratio of LDL2 particle number:LDL5 particle number, the ratio of the sum of LDL1 to LDL3 particle number:the sum of LDL4 to LDL4 particle number, the ratio of HDL1 particle number:HDL4 particle number and the ratio of the sum of HDL1 to HDL3 particle number:HDL4 particle number in the participants A) with cardiovascular disease (CVD), and B) with no CVD. (CVD, BMI 18.5-24.9 kg/m^2 n=14; CVD, BMI $\geq$30 kg/m^2 n=46; no CVD, BMI 18.5-24.9 kg/m^2 n=507; no CVD, BMI $\geq$30 kg/m^2 n=511).

[OFFICIAL

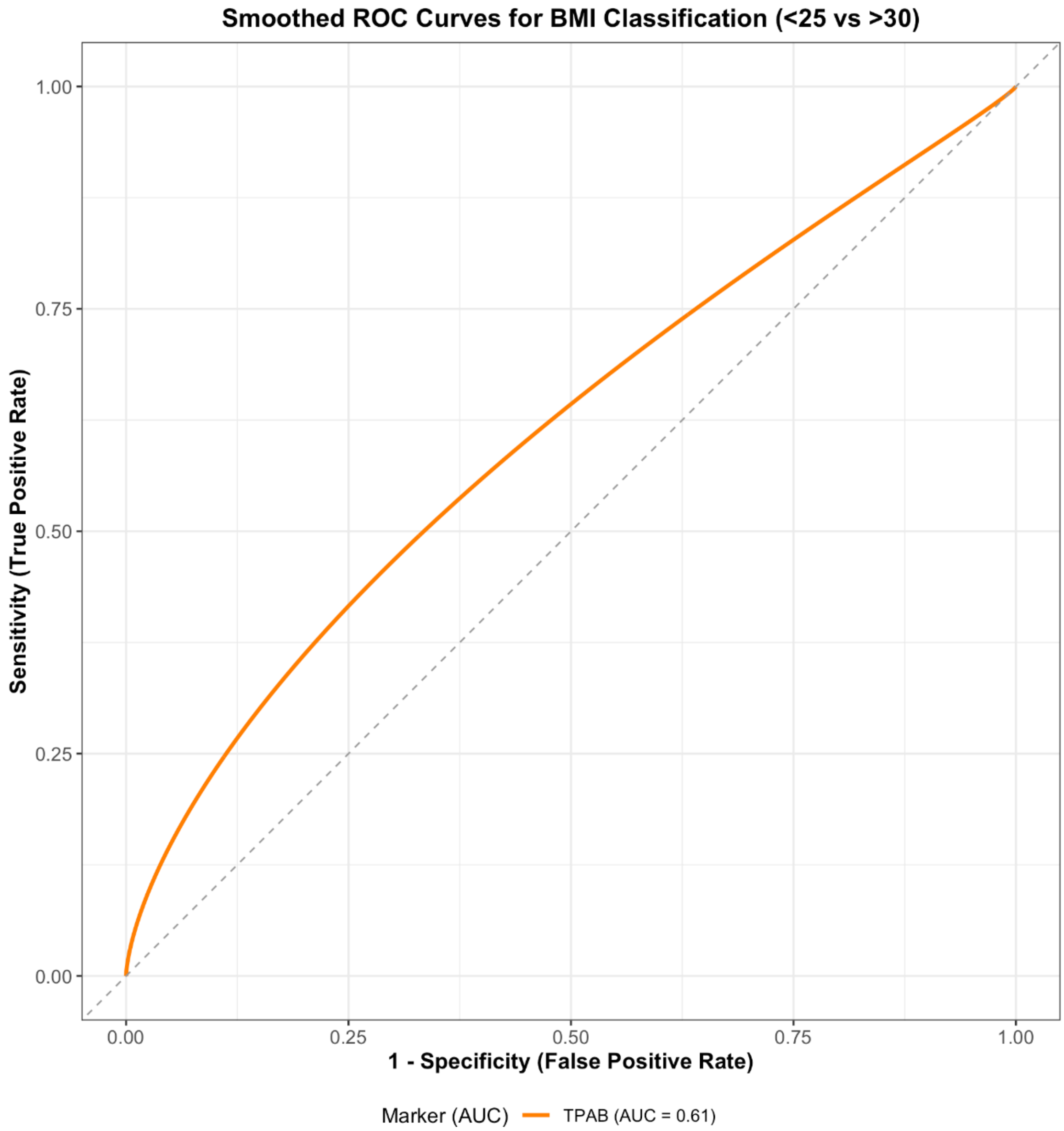

Figure S9. Area under the ROC curve for the healthy weight versus obese weight groups for the ApoB-100 particle number.

[OFFICIAL
